# *Clerodendrum chinense* Stem Extract and Nanoparticles: Effects on Proliferation, Colony Formation, Apoptosis Induction, Cell Cycle Arrest, and Mitochondrial Membrane Potential in Human Breast Adenocarcinoma Breast Cancer Cells

**DOI:** 10.3390/ijms25020978

**Published:** 2024-01-12

**Authors:** Chuda Chittasupho, Weerasak Samee, Mingkwan Na Takuathung, Siriporn Okonogi, Sathaporn Nimkulrat, Sirivan Athikomkulchai

**Affiliations:** 1 Department of Pharmaceutical Sciences, Faculty of Pharmacy, Chiang Mai University, Chiang Mai 50200, Thailand; chuda.c@cmu.ac.th (C.C.); siriporn.okonogi@cmu.ac.th (S.O.); 2 Center of Excellence in Pharmaceutical Nanotechnology, Faculty of Pharmacy, Chiang Mai University, Chiang Mai 50200, Thailand; 3 Department of Pharmaceutical Chemistry, Faculty of Pharmacy, Srinakharinwirot University, Nakhonnayok 26120, Thailand; weerasak@g.swu.ac.th; 4 Department of Pharmacology, Faculty of Medicine, Chiang Mai University, Chiang Mai 50200, Thailand; mingkwan.n@cmu.ac.th; 5 Clinical Research Center for Food and Herbal Product Trials and Development (CR-FAH), Chiang Mai 50200, Thailand; 6 Faculty of Pharmacy, Siam University, Bangkok 10160, Thailand; sathaporn.nim@siam.edu; 7 Department of Pharmacognosy, Faculty of Pharmacy, Srinakharinwirot University, Nakhonnayok 26120, Thailand

**Keywords:** anticancer, breast cancer, polyphenol, plant extract, nanoparticles

## Abstract

Breast cancer stands out as the most widespread form of cancer globally. In this study, the anticancer activities of *Clerodendrum chinense* (*C. chinense*) stem ethanolic extract were investigated. High-performance liquid chromatography (HPLC) analysis identified verbascoside and isoverbascoside as the major bioactive compounds in the *C. chinense* stem extract. Successfully developed nanoparticles exhibited favorable hydrodynamic diameter, polydispersity index, and surface charge, thus ensuring stability after four months of storage. The total phenolic content and total flavonoid contents in the nanoparticles were reported as 88.62% and 95.26%, respectively. The *C. chinense* stem extract demonstrated a dose-dependent inhibitory effect on MCF-7, HeLa, A549, and SKOV-3 cancer cell lines, with IC_50_ values of 109.2, 155.6, 206.9, and 423 µg/mL, respectively. *C. chinense* extract and NPs exhibited dose-dependent cytotoxicity and the highest selectivity index values against MCF-7 cells. A dose-dependent reduction in the colony formation of MCF-7 cells was observed following treatment with the extract and nanoparticles. The extract induced cytotoxicity in MCF-7 cells through apoptosis and necrosis. *C. chinense* stem extract and nanoparticles decreased mitochondrial membrane potential (MMP) and induced G0/G1 phase arrest in MCF-7 cells. In conclusion, use of *C. chinense* stem extract and nanoparticles may serve as a potential therapeutic approach for breast cancer, thus warranting further exploration.

## 1. Introduction

Cancer is a significant global health problem that affects millions of people worldwide, contributing to substantial morbidity, mortality, and economic burden. It is characterized by the uncontrolled growth and spread of abnormal cells in the body, leading to the formation of tumors and interference with normal bodily functions [1]. Breast cancer is the most common cancer in the world, and it continues to have a significant impact on the total number of cancer deaths. Global efforts are required to mitigate its growing burden, particularly in countries where its incidence is rapidly increasing, and mortality rates remain high. In 2020, there were over 2.3 million new cases of breast cancer and 685,000 deaths [2]. Despite increases in incidence over the last several decades, breast cancer mortality has decreased due to advancements in screening and targeted therapy [3]. Breast cancer is a genetic disease caused by mutations in neoplastic cells [4]. Mutations in tumor suppressor and oncogenic genes cause breast epithelial cells to develop a malignant phenotype [5]. These genetic changes influence breast cancer behavior, including responses to therapy and clinical outcomes.

The discovery of anticancer drugs from plants has played a pivotal role in the development of cancer treatments. Many successful anticancer drugs have originated from natural compounds found in plants. For example, vinca alkaloids, such as vinblastine and vincristine, derived from *Catharanthus roseus* have been used in the treatment of various cancers, including Hodgkin’s disease, choriocarcinoma, neuroblastoma, lymphosarcoma, and carcinomas of the breast and lungs [6]. While conventional anticancer drugs have revolutionized cancer treatment and improved patient outcomes, the rapid evolution of cancer cells and the development of resistance leads to ongoing challenges. As a result, the exploration of natural products, such as those found in plants, remains a promising approach for anticancer drug discovery.

Natural active compounds have garnered the interest of researchers due to their ability to deliver anticancer drugs with minimal toxicity [7]. Numerous herbs and their bioactive compounds have played an important role in the treatment of various types of cancer. Recent in vitro, in vivo, and clinical studies have revealed that a variety of bioactive compounds have the potential to be chemopreventive or chemotherapeutic agents against breast cancer. Various properties have been attributed to these natural bioactive compounds, including antiproliferative properties, induction of apoptosis, anti-inflammatory properties, antiangiogenic properties, anti-invasive and metastatic properties, cell cycle regulatory activity, tumor suppressor activities, and targeting of cancer stem cells against breast cancer [8].

Polyphenols exhibit cytotoxic effects on a variety of cancer cells [9]. Polyphenols exert their anticancer effects through diverse mechanisms, including the modification of signaling pathways, inhibition of cell cycle events, and induction of apoptosis, leading to the removal of cancer cells [10]. In addition, these compounds regulate the activities of enzymes pivotal in tumor cell proliferation [11]. Recent studies are increasingly implicating natural polyphenols in anticancer activities through a myriad of properties, including, but not limited to, antiangiogenic effects, antimetastatic properties, DNA interaction, and various other pathways [12,13,14].

*Clerodendrum* is a significant plant genus used as a traditional medicine to treat a variety of disorders. Several in vitro and in vivo tests have been conducted to validate these traditional claims. Various species of *Clerodendrum* genus have potent antimicrobial, anti-inflammatory, anti-malarial, anti-diabetic, anticancer, analgesic, and antioxidant properties [15]. Qi reported that the diterpene compound isolated from *C. chinense* root extract demonstrated anticancer activities against HL-60 and A-549 cell lines [16]. Barung et al. showed that ethanol extract, hexane fraction, ethyl acetate fraction, and water-soluble fraction of *C. chinense* had anticancer activity on A549 lung cancer cells [17]. The ethyl acetate fraction had the lowest IC_50_ value and was classified as moderately active against lung cancer. Previously, we reported the anticancer activities of *C. chinense* leaf and flower extracts against MCF-7, A549, and HeLa cells [18,19]. The leaf and flower extract of *C. chinense* have undergone extensive research in various cancer cell lines; however, there remains a gap in the existing knowledge, as the anticancer activities of the stem extract in relation to a breast cancer cell line have never been investigated.

Nanotechnology has emerged as an attractive research topic for drug delivery through the use of the designed nanomaterials in a variety of applications, such as pharmaceuticals and biomedicine. Nanoparticulate delivery systems aim to enhance the delivery of drugs or natural active compounds with poor water solubility. These features include facilitating site-specific targeting to minimize drug accumulation in healthy tissue, prolonging the presence of the drug in the body for effective treatment, extending drug bioactivity through protection from the biological environment, and enabling the transport of drugs across epithelial and endothelial barriers.

Recent advances in nanomaterials have paved the way for the development of novel cancer therapeutics. Due to their nanometer size, nanoparticles are considered an excellent option for passive and active targeted anticancer drug delivery [20]. In this investigation, we have used *C. chinense* stem extract for the first time to develop nanoparticles and determined its application for anticancer activity. *C. chinense* stem extract and NPs are expected to selectively target and affect cancer cells while preserving normal cells. The selectivity of these anticancer agents plays a pivotal role in mitigating the side effects associated with cancer treatment and has the potential to enhance therapeutic efficacy by concentrating on cancer cells. We hypothesized that *C. chinense* stem extract may exert anticancer activities through specific mechanisms and the nanoparticles of the extract can be used as a drug delivery system while preserving the anticancer activity of the extract.

## 2. Results

### 2.1. Yield of C. chinense Stem Extract

The extraction of *C. chinense* stem using 95% ethanol yielded 3.33 ± 0.58% *w*/*w*. The percentage yield indicated that approximately 3.33% of the soluble compounds in the *C. chinense* stem were successfully extracted using the maceration method. Several factors may have influenced the yield, including the temperature of extraction, extraction time, extraction solvent polarity, and the source of *C. chinense* stem [21]. The yield of *C. chinense* stem extraction was lower compared with *C. chinense* leaf (11.4%) and flower (11.2%) [18,19].

### 2.2. High-Performance Liquid Chromatography (HPLC) Profiles of Phytochemicals in C. chinense Stem Extract

**Figure 1:**
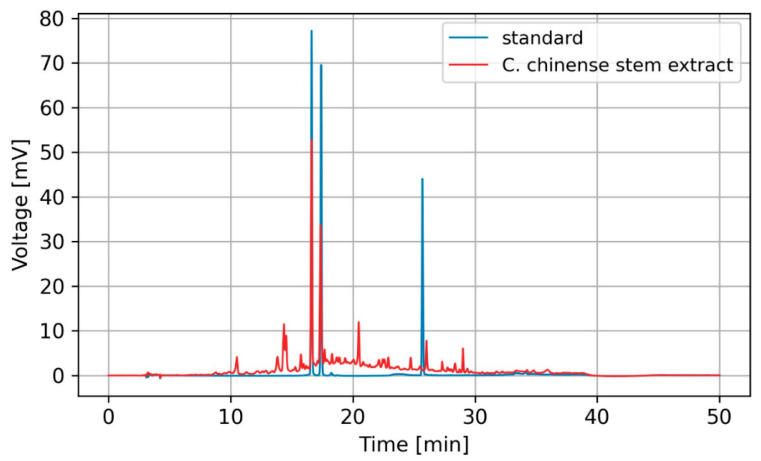
HPLC chromatogram of verbascoside, isoverbascoside, and hispidulin standards (blue) and *C. chinense* stem extract (red).

The HPLC analysis results revealed the presence of verbascoside and isoverbascoside as major bioactive compounds in *C. chinense* stem extract. Figure 1 shows representative HPLC chromatograms generated for the detection of two compounds. The results showed that *C. chinense* stem extract contained verbascoside and isoverbascoside. Hispidulin was not observed in this extract. Verbascoside was more abundant than isoverbascoside. Stem extract of *C. chinense* contained 9.42 ± 0.15 µg verbascoside per mg of extract and 8.32 ± 0.15 µg isoverbascoside per mg of extract. In our earlier investigations, we identified verbascoside, isoverbascoside, and hispidulin in the extracts from the leaves and flowers of *C. chinense*. Verbascoside exhibited higher abundance in the flower extract, whereas isoverbascoside was identified as a predominant compound in the leaf extract. The amount of verbascoside detected in the stem of *C. chinense* was lower compared with the flower (11.27 ± 0.08 µg/mg extract) [18]. The amounts of other compounds in the extract were too low to be identified or quantified through HPLC analysis and were neglected.

### 2.3. Characterization and Physical Stability Study of C. chinense Stem Extract Nanoparticles (NPs): Particle Size, Polydispersity Index (PDI), and Zeta Potential Value

**Figure 2:**
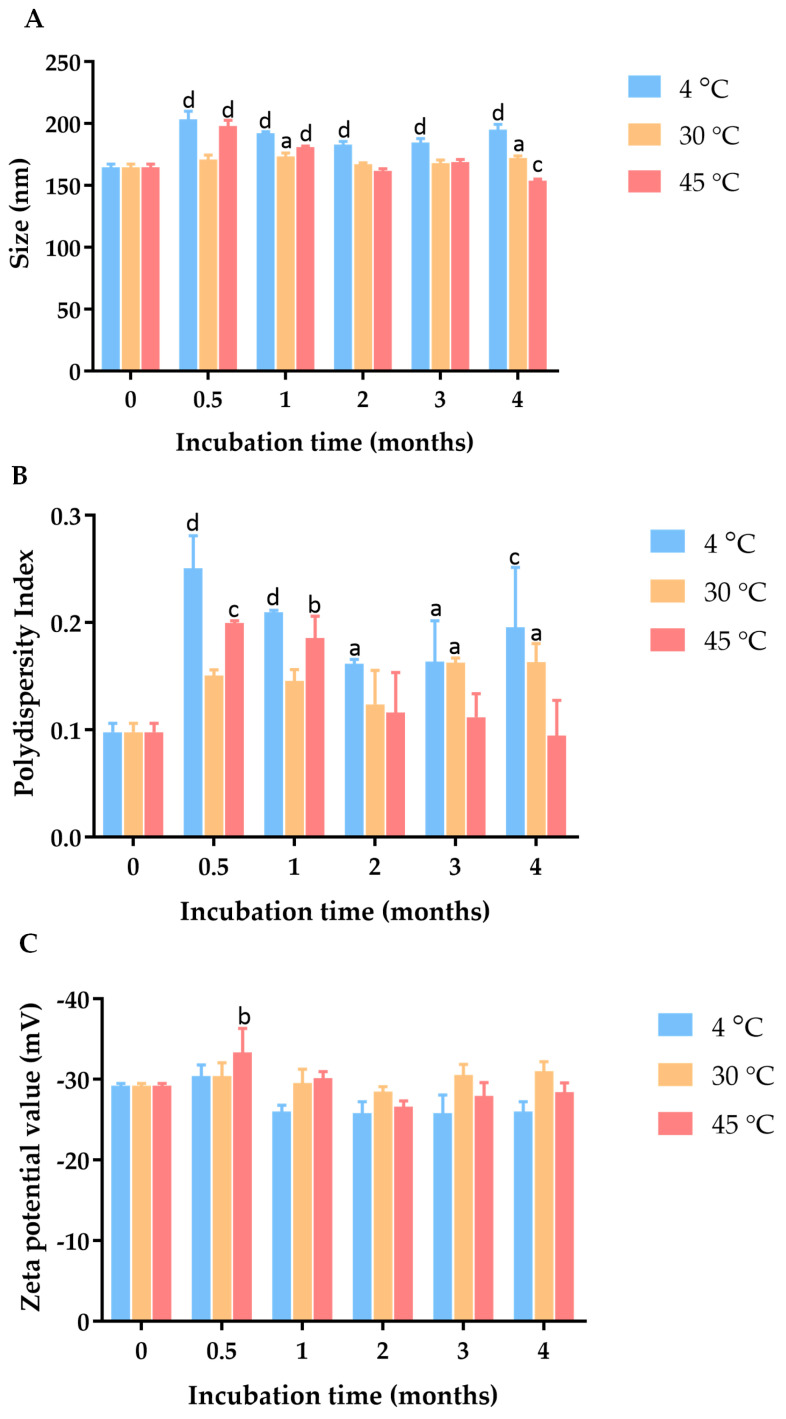
Effect of incubation time and temperature on (**A**) particle size, (**B**) PDI, and (**C**) zeta potential of *C. chinense* stem extract nanoparticles. Data are presented as the mean ± SD. The letters a, b, c, and d indicate *p* < 0.05, 0.01, 0.001, and 0.001, respectively. Tukey’s multiple comparisons test for size, polydispersity index, and zeta potential statistical analysis are shown in Supplementary Tables S1–S3.

**Figure 3:**
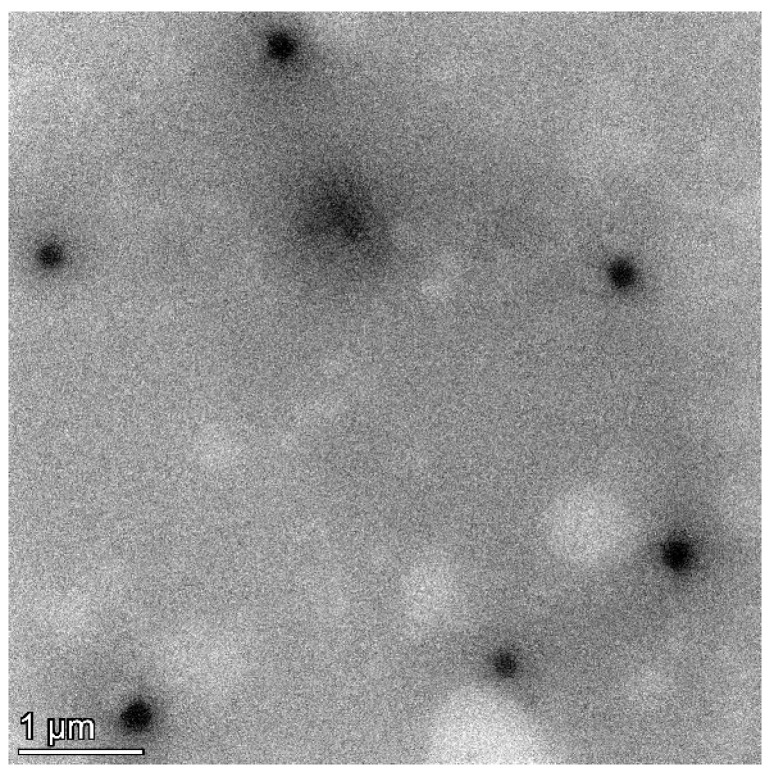
TEM image of *C. chinense* stem extract nanoparticles.

To determine the particle size, polydispersity index (PDI), and zeta potential, the *C. chinense* stem extract NPs were diluted tenfold with deionized water and subjected to analysis using a dynamic light-scattering-based particle size analyzer (Zetasizer Nano ZS, Malvern Instruments, Malvern, UK). Figure 2 and Supplementary Figure S1 revealed that the hydrodynamic diameter of *C. chinense* stem extract NPs measured 164.73 ± 2.37 nm. This size aligns closely with the findings from transmission electron microscopy (TEM) analysis, thereby confirming the accuracy of the results. The *C. chinense* stem extract NPs appear as spherical structures with a uniform size distribution (Figure 3). The NPs exhibited a relatively narrow size distribution, as evidenced by the low PDI value of 0.098 ± 0.008. The zeta potential of *C. chinense* stem extract NPs was determined to be −29.17 ± 0.32 mV (Supplementary Figure S2). These findings collectively demonstrate the successful application of the solvent displacement method in the preparation of *C. chinense* stem extract NPs.

To investigate the physical stability of these *C. chinense* stem extract NPs, samples were stored in a sealed container at three different temperature conditions, including 4 °C, 30 °C, and 45 °C, for a duration of 4 months. At specific intervals, particle size, PDI, and zeta potential measurements were conducted to monitor the influence of time and temperature on the NPs’ physical properties. The hydrodynamic diameter and polydispersity index of the *C. chinense* extract NPs were assessed at various time points during the stability study (Supplementary Figure S3). After four weeks of storage at 4 °C, 30 °C, and 45 °C, the maximum average particle sizes of NPs were 203.47 ± 6.39 nm, 173.30 ± 2.77 nm, and 197.80 ± 4.87 nm, respectively. The PDI values of NPs were 0.25 ± 0.03, 0.16 ± 0.02, and 0.20 ± 0.00 when stored at 4 °C, 30 °C, and 45 °C, respectively. These results suggested that there was minimal and acceptable change in particle size and size distribution during this period. Zeta potential measurements were conducted to assess the surface charge of the nanoparticles, which can influence their stability. The results showed that the zeta potential ranged between −29.2 mV and −33.33 mV at 4 °C, 30 °C, and 45 °C, suggesting that the surface charge was relatively maintained, thus contributing to the stability of the nanoparticles.

### 2.4. Total Phenolic Content and Total Flavonoid Content in C. chinense Stem Extract and NPs

The total phenolic contents in *C. chinense* stem extract and NPs were found to be 553.20 ± 68.74 and 490.26 ± 24.12 milligrams of gallic acid equivalent (GAE) per gram of extract, respectively. The total flavonoid compounds in *C. chinense* stem extract and NPs were expressed in 185.44 ± 37.39 and 176.65 ± 13.07 milligrams of quercetin equivalent (QE) per gram of extract. These results revealed that the total phenolic content and total flavonoid contents in the *C. chinense* stem extract NPs were 88.62% and 95.26%, respectively. The strong correlations between NP concentrations and TPC and TFC are shown in Supplementary Figures S4 and S5.

Among different parts of the plants, ethanolic extract of *C. chinense* leaf offered the highest TPC (1.03 g GAE/g extract), followed by stem extract (0.553 g GAE/g extract) and flower extract (0.487 g GAE/g extract) [18,19]. The results of total flavonoid content analysis showed that the stem of *C. chinense* exhibited the lowest concentration of total flavonoid content, followed by the leaf and flower [18,19]. These results indicated that each part of *C. chinense* contained different amounts of phenolic compounds and flavonoids.

### 2.5. Antioxidant Activities of C. chinense Stem Extract Nanoparticles and NPs

**Figure 4:**
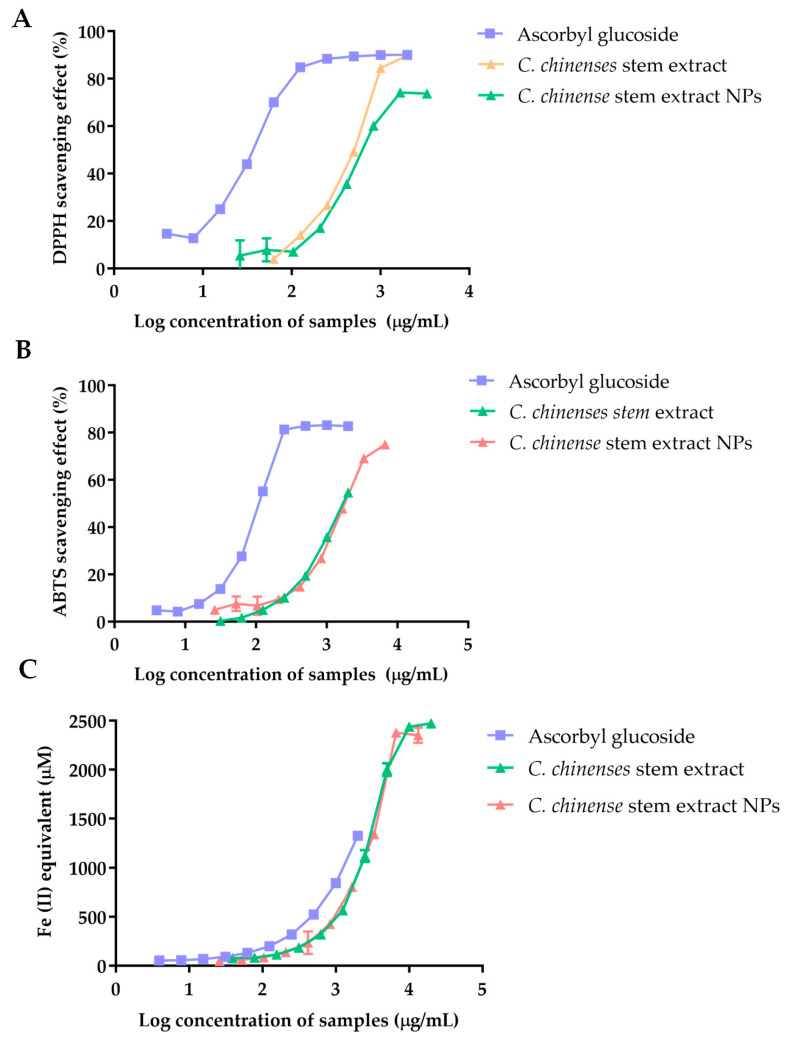
The antioxidant activity of ascorbyl glucoside, *C. chinense* ethanolic stem extract, and NPs determined using (**A**) a DPPH free radical scavenging assay, (**B**) an ABTS free radical scavenging assay, and (**C**) a FRAP assay.

Figure 4 illustrates the 2,2-diphenyl-1-picrylhydrazyl (DPPH) scavenging activity of ascorbyl glucoside, the extract from *C. chinense* stems, and NPs. The scavenging ability against DPPH radicals was expressed as a percentage (%), and the results demonstrated a concentration-dependent relationship. A plateau effect was observed at higher concentrations, indicating the saturation of the free radical scavenging. The IC_50_ values were calculated as 35.71 µg/mL for ascorbyl glucoside, 457.1 µg/mL for the *C. chinense* stem extract, and 724.9 µg/mL for *C. chinense* stem extract NPs. The results suggested that the extract exhibited greater DPPH radical scavenging activity when compared to the NPs.

2,2′-Azino-bis(3-ethylbenzothiazoline-6-sulfonic acid) (ABTS) radical scavenging activity of ascorbyl glucoside, *C. chinense* stem extract, and *C. chinense* stem extract NPs was correlated with the concentration of the samples. The higher ABTS radical scavenging activity was observed in *C. chinense* stem extract compared to its NPs. The IC_50_ values were reported at 117.2 µg/mL, 1688 µg/mL, and 1892 µg/mL for ascorbyl glucoside, extract, and NPs, respectively.

The reducing activity of ascorbyl glucoside, *C. chinense* stem extract, and NPs was measured using Ferric Reducing Antioxidant Power (FRAP) assay. Ascorbyl glucoside at a concentration range of 3.9–2000 µg/mL had a FRAP value of 52.52 ± 1.25 to 1324.48 ± 8.84 µM. The FRAP values of *C. chinense* stem extract and NPs were observed at 76.96 ± 2.93 to 2471.94 ± 15.16 µM and 48.04 ± 3.95 to 2350.52 ± 77.74 µM, respectively. It was found that the ferric reducing power of all samples depended on the concentration of the samples. A previous report showed that FRAP values of *C. chinense* leaf extract were in the range of 88.73 ± 4.59 to 2480.81 ± 0.00 µM. Therefore, *C. chinense* stem extract showed lower FRAP values compared with leaf extract. The lower antioxidant activities of *C. chinense* stem extract NPs might be due to the undissolved extract encapsulated in the NPs.

Deb and Sarkar have shown that the total polyphenol and verbascoside contents were directly proportional to the antioxidant potential of *Clerodendrum glandulosum* Lindl. leaves [22]. Funes et al. also reported the correlation between verbascoside in *Aloysia triphylla* and the significant ferric-reducing ability of plasma of this compound [23]. Therefore, it might be concluded that the ferric-reducing potential of *C. chinense* stem extract might be partly due to verbascoside.

The correlations between the total phenolic content and the total flavonoid content and the antioxidant activities of *C. chinense* stem extract and the NPs were calculated using the Pearson correlation coefficient. The results of the correlation are shown in Table 1.

### 2.6. Cytotoxicity of C. chinense Stem Extract and NPs against Cancer Cell Lines

**Figure 5:**
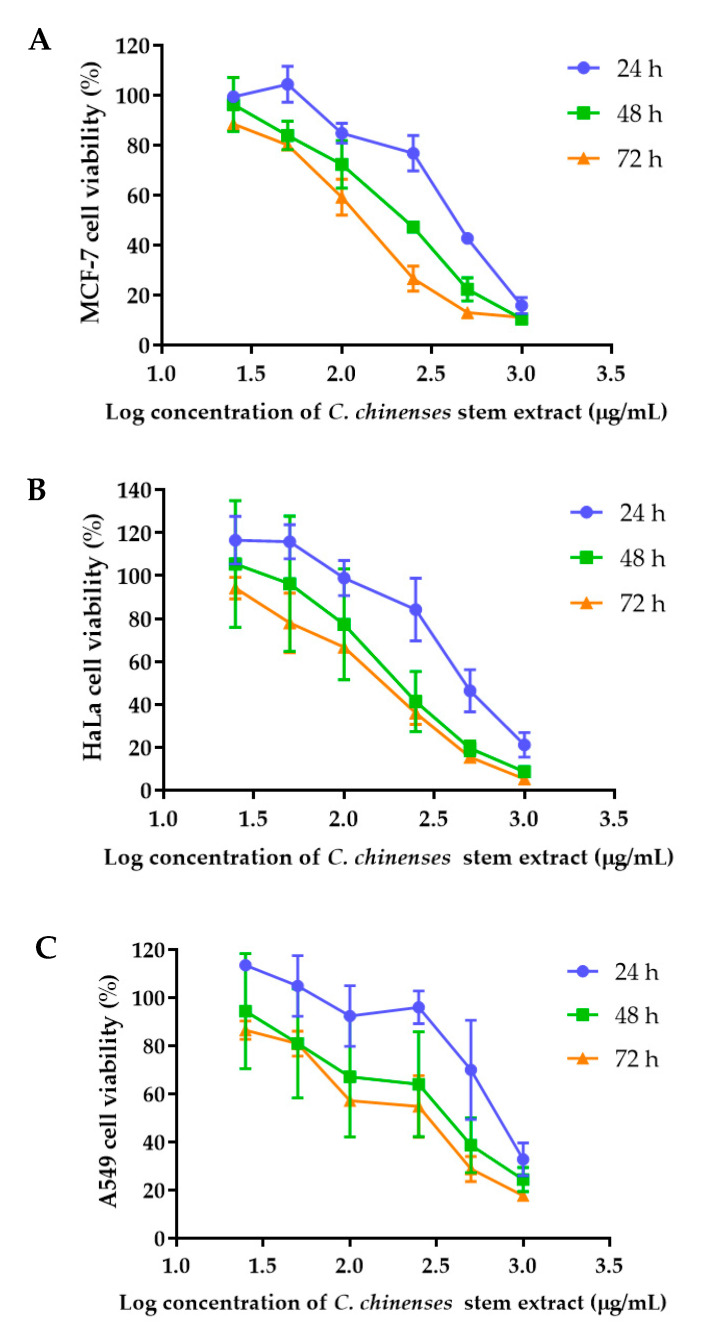

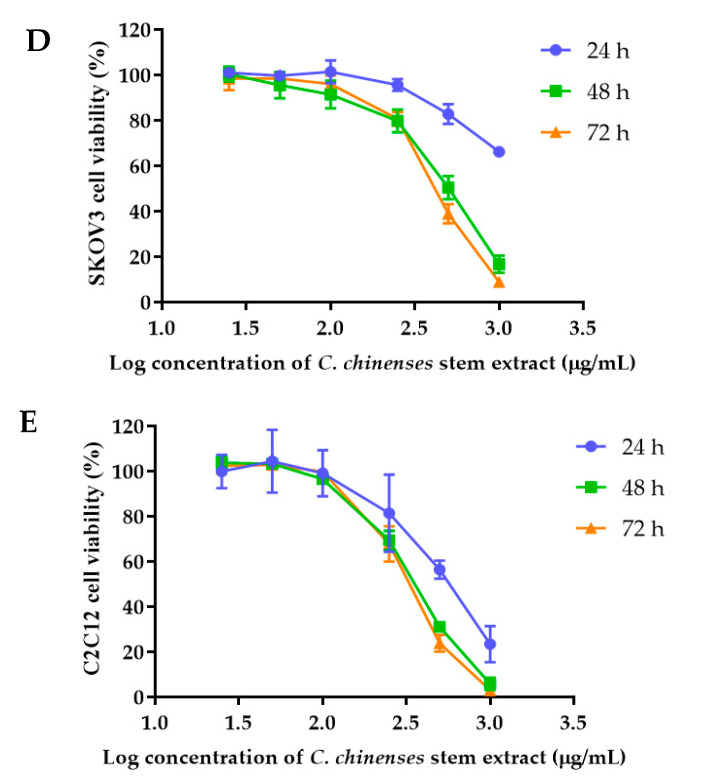
Effect of *C. chinense* stem extract on viability and growth of (**A**) MCF-7 breast cancer cells, (**B**) HeLa cervical cancer cells, (**C**) A549 lung cancer cells, (**D**) SKVO-3 ovarian cancer cells, and (**E**) C2LC12 myoblast cells. Data are presented as the mean ± SD of the three independent experiments.

**Figure 6:**
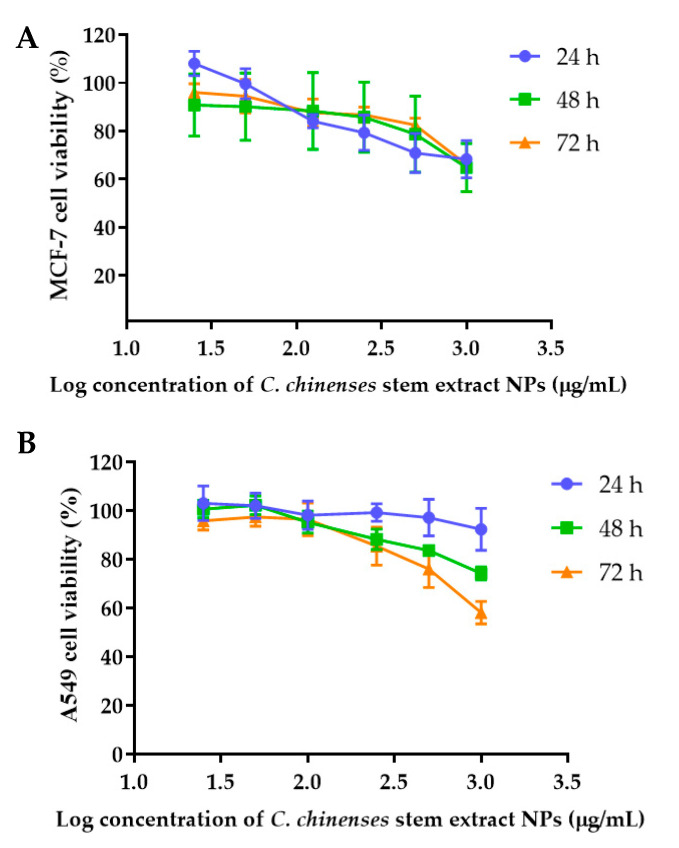

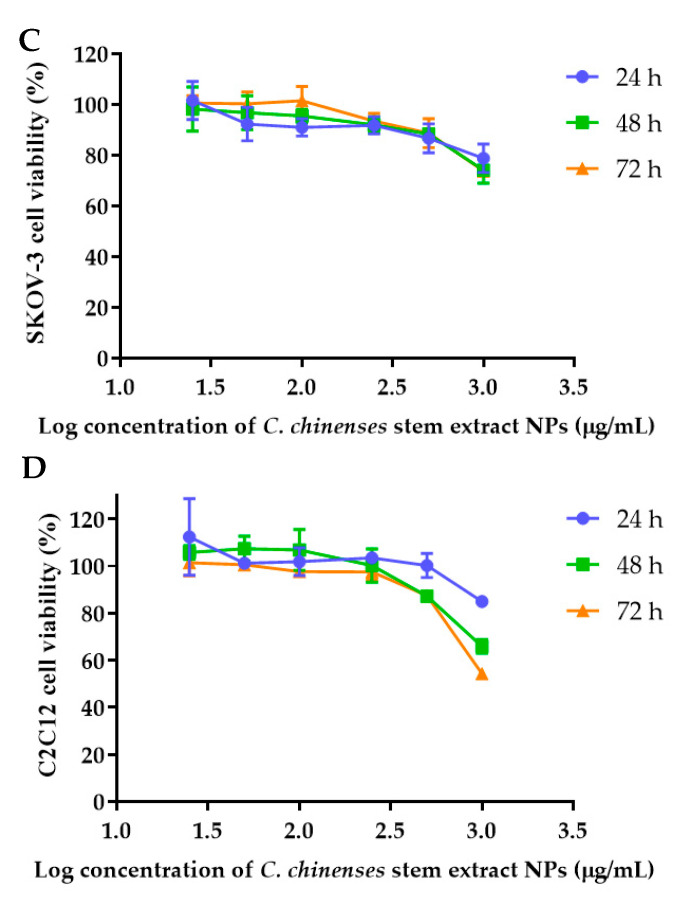
Effect of *C. chinense* stem extract NPs on viability and growth of (**A**) MCF-7 breast cancer cells, (**B**) A549 lung cancer cells, (**C**) SKVO-3 ovarian cancer cells, and (**D**) C2CL12 myoblast cells. Data are presented as the mean ± SD of the three independent experiments.

The anticancer activities of the *C. chinense* stem extract were tested for the first time in our study. To confirm the anticancer activity of the *C. chinense* stem extract, the cytotoxicity of the extract was tested in several cancer cell lines, including HeLa, A549, MCF-7, and SKOV-3 cells. The most sensitive cell line to this extract was further investigated for the anticancer activity mechanisms. *C. chinense* stem extracts exhibited a dose-dependent growth-inhibitory effect on the MCF-7 breast cancer cell line, the HeLa cervical cancer cell line, the A549 adenocarcinomic human alveolar basal epithelial cells, and the SKOV-3 ovarian cancer cells (Figure 5). *C. chinense* stem extract showed the lowest IC_50_ values against the MCF-7 breast cancer cell line compared with HeLa cells, A549, and SKOV-3 cells. The ethanol extract of *C. chinense* stem showed the most potent inhibitory effect with IC_50_ values of 109.2, 155.6, 206.9, and 2044 µg/mL on the MCF-7, HeLa, A549, and SKOV-3 cell lines, respectively. The MCF-7 growth-inhibitory effects were also detected in the ethanolic extract of *C. chinense* flower, with an IC_50_ value of 67.05 µg/mL. The lower inhibitory effect of most of the stem extracts compared with the flower extract might be due to the absence of hispidulin, which is considered one of the bioactive compounds in the *C. chinense* extract. NPs of *C. chinense* stem extract have demonstrated cytotoxicity against MCF-7, A549, and SKOV-3 cells (Figure 6). However, the cell-growth-inhibitory effect of the NPs encapsulating the extract was lower compared with the extract itself. The cytotoxicity of *C. chinense* stem extract and NPs was also investigated in normal myoblast cells, namely C2C12, to investigate the selectivity of the extract and the NPs. The cytotoxicity of both the extract and the NPs against C2C12 was found to be dose dependent. Both the extract and the NPs were less toxic to C2C12 cells compared to MCF-7 and A549 cells. Micrographs of A549, SKOV-3, and C2C12 cells treated with *C. chinense* stem extract or NPs at 1 mg/mL are shown in Supplementary Figure S6.

The selectivity index of *C. chinense* stem extract and NPs, calculated by dividing the IC_50_ value of samples against noncancerous cells (C2C12) with that of samples against cancerous cells (MCF-7, HeLa, A549, and SKOV-3 cells), is shown in Table 2. It was found that *C. chinense* stem extract had the highest selectivity index values against MCF-7 cells compared with HeLa, A549, and SKOV-3 cells. Therefore, *C. chinense* stem extract not only showed the highest cytotoxicity against the MCF-7 cell line but also the highest selectivity to this cell line. For this reason, we further developed the NPs of *C. chinense* stem extract and tested their toxicity and selectivity index on MCF-7 cells. The results were confirmed by using A549 and SKOV-3 cells. The NPs of *C. chinense* stem extract have been shown to be preferably selectively toxic to MCF-7 and A549 cells as opposed to C2C12 cell lines. The %cell viability of MCF-7 and C2C12 after exposure to the extract and the NPs were compared at each time point. The results are shown in Supplementary Figure S7. NPs had lower IC_50_ values against C2C12 cells compared with the SKOV-3 cancer cell line, suggesting that *C. chinense* stem extract NPs are not selective to this type of cancer cell line. The selectivity index was greatest when cells were incubated with NPs for 24 h and decreased after incubation for 48 and 72 h. These results suggested that as the incubation time increased, the NPs may exhibit more toxicity towards healthy cells or tissues.

### 2.7. C. chinense Stem Extract and NPs Inhibited Colony Formation of MCF-7 Cells

**Figure 7:**
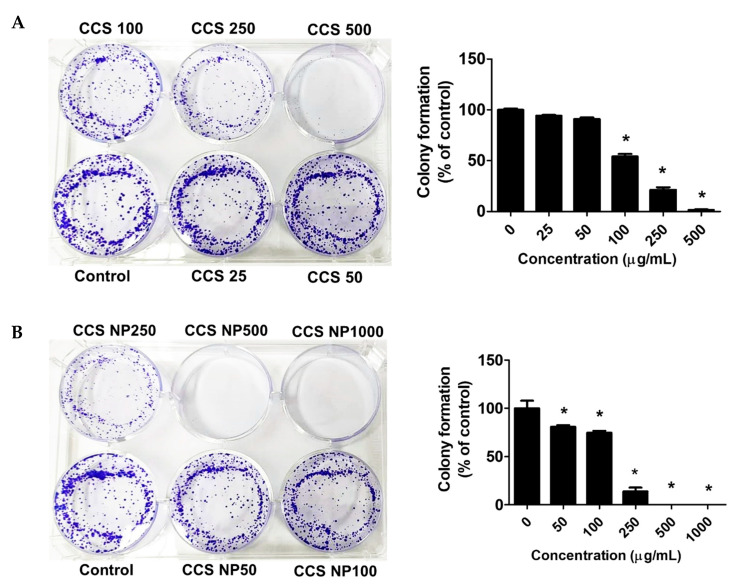
Effects of (**A**) *C. chinense* stem extract and (**B**) NPs on colony formation of the MCF-7 cancer cell lines. Data are presented as the mean ± SD of the three independent experiments. * indicates *p* < 0.05. Tukey’s multiple comparisons test for colony formation percentage statistical analysis are shown in Supplementary Table S4.

The antiproliferative activity of the extract was further evaluated using the clonogenic survival assay. Colony formation assays suggested that the colony-forming ability of the MCF-7 cells was remarkably reduced in a dose-dependent manner after treatment with *C. chinense* stem extract and NPs for 24 h (Figure 7). MCF-7 cells formed 100.00 ± 1.99% without treatment. MCF-7 cells treated with 25, 50, 100, 250, and 500 µg/mL *C. chinense* stem extract showed 94.05 ± 1.60%, 90.91 ± 2.69%, 54.02 ± 4.48%, 21.33 ± 4.21%, and 1.40 ± 1.09% colony formation, respectively, compared with the control. MCF-7 cells treated with 50, 100, and 250 µg/mL NPs showed 80.81 ± 3.08%, 74.81 ± 3.69%, and 13.76 ± 6.94% colony formation, respectively, compared with the control. When treated with 500 and 1000 μg/mL of NPs, all of the cells were unable to form visible colonies. The ability of *C. chinense* stem extract and NPs to affect long-term colony formation may be in part through both suppression and/or induction of cell death [24]. *C. chinense* stem extract at 500 µg/mL reduced MCF-7 colony formation to 1.40 ± 1.09%, while NPs at the same concentration completely inhibited cancer cell colony formation.

### 2.8. C. chinense Stem Extract and NPs Promoted Apoptosis in MCF-7 Cells

**Figure 8:**
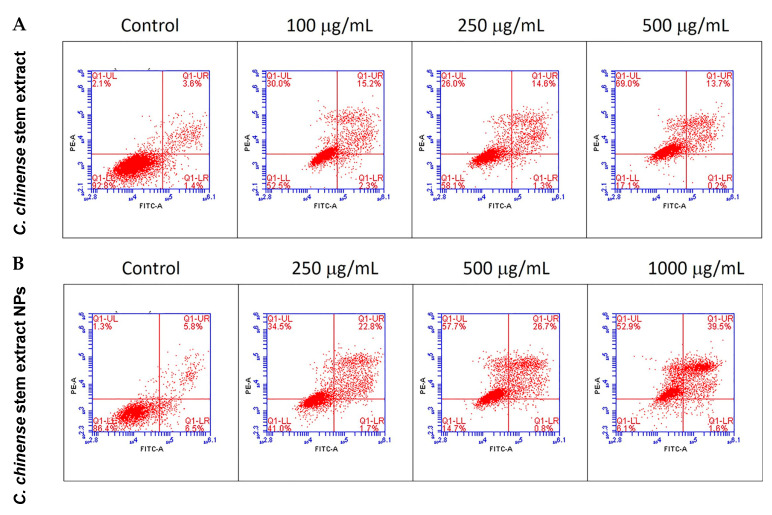
Flow cytometry analysis with Annexin V-PI staining was performed to evaluate the percentage of apoptotic cells in (**A**) *C. chinense* stem extract and (**B**) NP-induced MCF-7 cells.

The apoptotic effect of the *C. chinense* stem ethanolic extract was investigated by staining the cells with the Annexin V-FITC apoptosis detection kit and evaluation through flow cytometry. Viable cells were identified as those negative for both propidium iodide (PI) and annexin V. Early apoptotic cells were characterized as PI negative with annexin V staining and reduced cell size. Necrotic cells were identified as PI-positive cells with either normal or enlarged cell size. Late apoptotic cells were defined as PI-positive cells exhibiting annexin V staining and diminished cell size [25]. The results showed that *C. chinense* stem extract and NPs induced MCF-7 cytotoxicity through the mechanisms associated with apoptosis and necrosis in a concentration-dependent manner (Table 3, Figure 8). The late apoptosis and necrosis of the negative control group were 3.3–5.7% and 1.27–2.17%, respectively. About 14.07%, 14.77%, and 12.13% of the MCF-7 cells experienced late apoptosis at 100, 250, and 500 µg/mL of the *C. chinense* stem extract, while the NPs induced 26.73%, 25.57%, and 40.13% of late-apoptotic MCF-7 cells at 250, 500, and 1000 µg/mL, respectively.

### 2.9. C. chinense Stem Extract and NPs Decreased Mitochondrial Membrane Potential (MMP)

**Figure 9:**
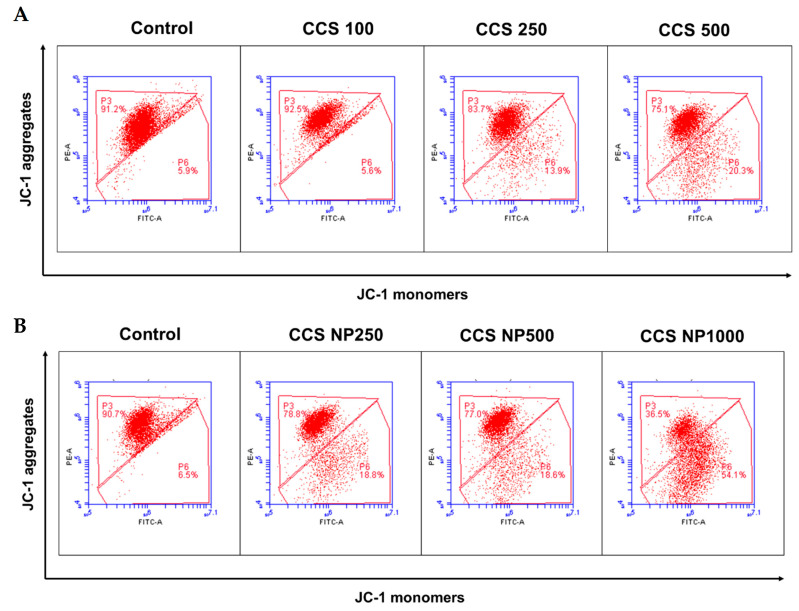
Flow cytometry analysis of the JC-1 assay (mitochondrial membrane potential) in MCF-7 cells treated with (**A**) *C. chinense* stem extract and (**B**) NPs.

Flow cytometry analysis of 5,5,6,6-tetrachloro-1,1,3,3-tetraethylbenzimidazolylcarbocyanine iodide (JC-1) staining demonstrated a significantly decreased fluorescence intensity of aggregates and an increased fluorescence intensity of monomers (Figure 9). MCF-7 cells exposed to *C. chinense* stem extract at 500 µg/mL and NPs at 250 and 500 µg/mL showed a significantly higher percentage of JC-1 monomers compared to the control group (Table 4). The ratio of JC-1 aggregates to JC-1 monomers decreased with the concentration of *C. chinense* stem extract and NPs. The ratio of monomers and aggregates significantly increased from 0.066 in the control group to 0.20 in the extract at 500 µg/mL in the treated group and to 1.38 in NPs in the 1000 µg/mL group, indicating a dramatic reduction in MMP and resultant mitochondrial dysfunction in the *C. chinense* extract and NP-treated groups. These results suggested that the mitochondrial dysfunction that resulted from the extract and NPs was dose dependent. The ratios of monomers to aggregates were increased when cells were treated with NPs compared to the extract at all tested concentrations. Ratios of monomers to aggregates when cells were treated with 250 and 500 µg/mL extract were 0.13 and 0.20, respectively. NPs at 250 and 500 µg/mL increased ratios of monomers to aggregates to 0.185 and 0.233, respectively. This result suggested that NPs decreased mitochondrial membrane potential to a greater extent compared with the extract.

### 2.10. C. chinense Stem Extract and NP-Induced Cell Cycle Arrest

**Figure 10:**
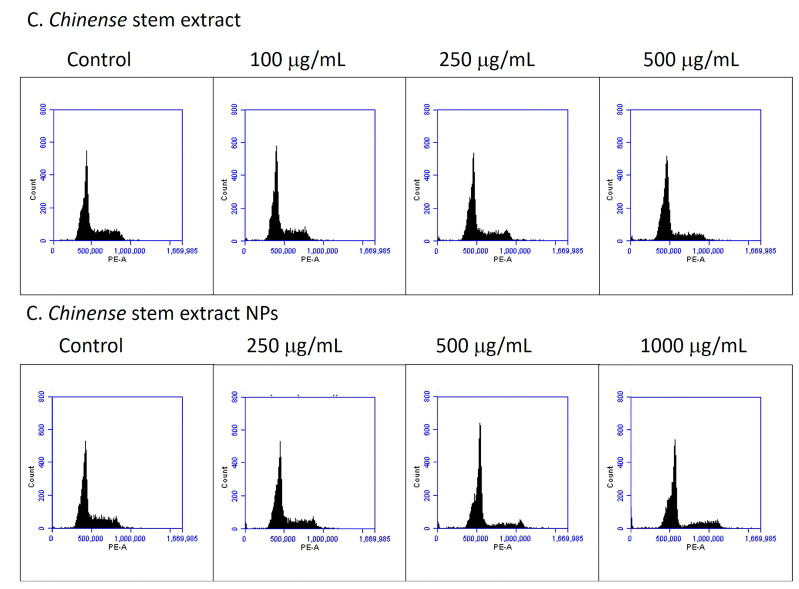
*C. chinense* stem extract and NPs induce G0/G1 phase cell cycle arrest.

Cell cycle distribution was analyzed to further investigate the potential mechanism by which *C. chinense* stem extract and NPs depressed MCF-7 cell growth. MCF-7 cell lines were treated with *C. chinense* stem extract and NPs for 24 h followed by flow cytometry assays. The representative cell cycle distributions of MCF-7 cells exposed to *C. chinense* stem extract and NPs are presented in Figure 10. The results show that *C. chinense* stem extract caused G0/G1 phase arrest in MCF-7 cells at a concentration of 500 µg/mL (Table 5). In particular, the proportion of G0/G1 phase cells significantly increased as the concentration of the extract increased, which was accompanied by a decrease in the number of cells in the S phase and the G2/M phase. Therefore, the effect of *C. chinense* stem extract and NPs on the proliferation of MCF-7 cells is likely associated with cell cycle arrest.

## 3. Discussion

The yield of stem extraction was significantly lower than that of the flower and leaves of *C. chinense* when the same solvent and extraction method were used for extraction. However, the bioactive compound contents in the stem extract were comparable to those of the flower extract. The stem extract contained a lower amount of 9.42 ± 0.15 µg verbascoside/mg of extract, while the flower extract contained 11.27 ± 0.08 µg/mg of extract. Hispidulin was negligible in the stem extract, while the flower extract contained a low amount of hispidulin (0.98 ± 0.01 µg/mg extract). Isoverbascoside content was higher in the stem compared with the flower extract (8.32 ± 0.15 µg/mg extract vs. 4.30 ± 0.27 µg/mg extract). The cytotoxicity investigations demonstrated that the flower extract exhibited lower IC_50_ values compared to the stem extract, signifying greater cytotoxicity in the flower extract. These findings imply a correlation between yield, bioactive compounds, and cytotoxicity.

The antioxidant activity observed in the *C. chinense* extract and NPs may be attributed to the presence of phenolic acids and flavonoids. The correlations between TPC and TFC and the antioxidant activities of *C. chinense* stem extract and NPs were calculated using the Pearson correlation coefficient. The results of the correlation are shown in Table 1. Pearson correlation coefficients revealed that the antioxidant activity of *C. chinense* extract NPs depended on the total phenolic and flavonoid contents.

The DDPH and ABTS free radical scavenging activity and the ferric-reducing power of the *C. chinense* extract correlated with the total phenolic content, whereas only the ABTS free radical scavenging activity and ferric-reducing power of the *C. chinense* extract correlated with the total flavonoid content. These natural phenolic compounds are recognized for their antioxidant properties, which can mitigate the risk of cancer diseases associated with oxidative stress. It is noteworthy that certain phenolic compounds, including phenolic acids, flavonoids, and non-flavonoids, can exhibit prooxidant behavior under specific conditions [26]. A high concentration of phenolic compounds and elevated pH levels may contribute to their prooxidant activity, leading to the induction of apoptosis and necrosis in cancer cells [27]. Hence, it is crucial to note that the behavior of phenolic compounds as either antioxidants or prooxidants depends on the redox homeostasis of the cells.

In this study, it was observed that the *C. chinense* stem extract NPs exhibited inhibitory effects on the growth of MCF-7 cells at concentrations of 500 and 1000 μg/mL after 24 h of incubation. The cytotoxicity of the NPs on MCF-7 breast cancer cells was found to be dose dependent. The IC_50_ values of the NPs were consistently higher than those of the extract across all incubation time points. Compared with the NPs, the %cell viability of the 1 mg/mL extract after 72 h of incubation was six-fold. These findings imply that the poloxamer 407 used to stabilize the NPs might mitigate the toxicity of the extract, possibly by delaying its release to the cells.

The cytotoxicity and antiproliferative effects of the *C. chinense* stem extract and NPs might result from verbascoside, as it has been shown that verbascoside has a cytotoxic effect on MCF-7 breast cancer cells. Senol er al. reported that the IC_50_ values for the MCF-7 BC cell line after 24, 48, and 72 h of exposure to a different concentration of verbascoside were 0.127, 0.2174, and 0.2828 μM, respectively [28]. Verbascoside isolated from *Scrophularia subaphylla* L. exhibited cytotoxicity on MCF-7 cells, and the IC_50_ value was found to be 0.39 ± 0.015 μg/mL after 48 h of exposure [29]. Verbascoside significantly inhibits colorectal cancer cell growth in vivo, represses cell proliferation, and promotes apoptosis by modulating the HIPK2-p53 signaling pathway [30]. Isoverbascoside caused a G0/G1 cell cycle arrest, thereby inhibiting the cell proliferation of MGC 803 gastric cancer [31].

*C. chinense* stem extract NPs demonstrated better MCF-7 cell colony formation inhibition compared with the extract. This effect might be due to the released bioactive compounds during 15 days of incubation. Several studies have reported the effects of phenolic compounds on cancer cell colony formation. Zhang et al. showed that different doses of glycyrrhizinic acid had a tendency to inhibit or suppress the colony formation tendency of these MCF-7 breast cancer cells [32]. Epigallocatechin gallate (EGCG) was shown to inhibit colony formation and induce apoptosis of MCF-7 breast cancer cells via down-regulation of survivin, a major member of the IAP gene family [24].

The results indicated that at 500 µg/mL, stem extract can induce 12.13% of late-apoptotic cells, while NPs induced 25.57% of apoptotic cells. The enhancement of apoptosis induction by NPs encapsulating *C. chinense* stem extracts can be attributed to several factors, including enhancing the bioavailability of bioactive compounds present in the extract. The NPs may improve the solubility and stability of the bioactive compounds, thus providing better absorption by the cells. This increased bioavailability can lead to more effective interactions with cellular components, promoting apoptosis. NPs can facilitate the cellular uptake of *C. chinense* stem extract. The small size and unique surface properties of nanoparticles may enhance their interaction with cell membranes, thus promoting internalization of the encapsulated or surface-bound bioactive substances.

MMP serves as a key indicator of mitochondrial activity, and a decline in MMP is often indicative of mitochondrial dysfunction. To investigate MMP alterations, we utilized JC-1, a dye specifically designed for mitochondria. JC-1 exhibits red fluorescence when forming aggregates in healthy mitochondria. As the membrane potential decreases, JC-1 transitions into monomers, thus resulting in green fluorescence. The alteration in the ratio of red to green fluorescence serves as an indicator of the mitochondrial condition under examination. The results showed that the *C. chinense* stem extract and the NPs can decrease the mitochondrial membrane potential in a concentration-dependent manner.

Many studies have demonstrated that polyphenols induce apoptosis and dissipate mitochondrial membrane potential in cultured cancer cells. Curcumin induced apoptosis in human gastric adenocarcinoma SGC-7901 cells through dissipation of the mitochondrial membrane potential [33]. Honokiol induced apoptosis and decreased mitochondrial membrane potential of neuroblastoma neuro-2a cells at 40 μM exposure [34]. EGCG inhibited HeLa cervical cancer cells in a concentration- and time-dependent manner by inducing apoptosis, including a decrease in the mitochondrial membrane potential [35]. Qanungo et al. reported that EGCG can indeed promote apoptosis in pancreatic cancer through mitochondrial membrane depolarization [36]. Polyphenols extracted from pinecones of *Pinus koraiensis* inhibited tumor growth in sarcoma-bearing 180 mice by activating the mitochondrial apoptotic pathway [37].

Both *C. chinense* stem extract and NPs at all tested concentrations significantly induced cell cycle arrest at the G0/G1 phase. At 500 µg/mL, the *C. chinense* stem extract NPs increased cells at the G0/G1 phase by 74.63%, which was higher than the extract (71.40 ± 0.56%). The results suggested that the component in NPs did not affect the cell cycle arrest of the extract. Many studies have showed that polyphenolic compounds can suppress the cell cycle. Analysis of a polyphenol-enriched extract from selenium-enriched Ziyang green tea (ZTP) suggested that the treated cells were subjected to a blockage at the G1 phase of the cell cycle, suggesting that ZTP blocked the progression of the cell cycle at the G0/G1 phase by modifying p53 and CDK2 expression [38]. A polyphenol-enriched extract obtained from *Salvia chinensis* induced G0/G1 cell cycle arrest and induced a significant and concentration-dependent reduction in the mitochondria membrane potential of the MiapaCa-2 human pancreatic cancer cells [39].

The anticancer activity of *C. chinense* stem extract may result from verbascoside and isoverbascoside. The anticancer activity of verbascoside has been reported. Verbascoside is associated with cell cycle arrest and apoptosis of breast cancer cells, including MCF-7 and MDA-MB-231 cells. Verbascoside exhibited a dose-dependent impact on the modulation of cell cycle and apoptosis-related proteins. The levels of CyclinB1, Cdc2, Bcl-2, and survivin were reduced, whereas cleaved PARP1, BAX, and cleaved caspase3/9 increased. Mechanistically, verbascoside inhibited the PI3K/AKT signaling pathway [40]. In another study, verbascoside elevated the population of subG1 cells and increased cell apoptosis rates. Additionally, it demonstrated time-dependent generation of reactive oxygen species (ROS) in tumor cells within 1–24 h of incubation [41].

## 4. Materials and Methods

### 4.1. Preparation of C. chinense Stem Extract

**Figure 11:**
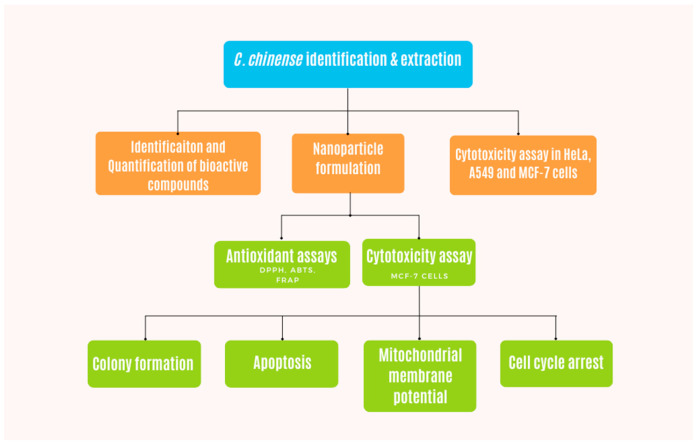
Flow chart demonstrating the methodology of this work.

**Figure 12:**
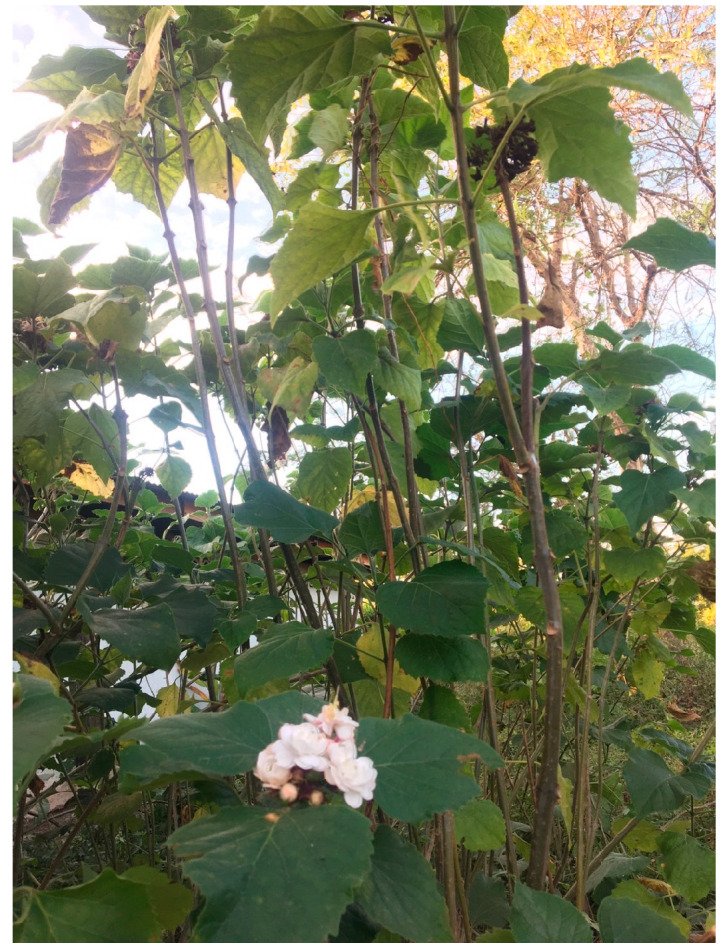
*C. chinense* shrub.

Figure 11 demonstrates the flow chart of experiments performed with *C. chinense* extract in this study. Stems of *C. chinense* were collected from Chiang Mai, Thailand (Figure 12). The plant was identified by Assistant Professor Sirivan Athikomkulchai with a voucher specimen named SIRA003. The voucher specimen was stored at the Faculty of Pharmacy, Srinakharinwirot University, Nakhonnayok, Thailand. The stems of *C. chinense* were thoroughly washed with water and dried at room temperature. Then, the stems were ground to fine powder using a kitchen blender. Next, 10 g of the stem powder was mixed with 100 mL of 95% ethanol. The extract was filtered with Whatman filter paper No 1. The filtrate extract was dried to a crude extract using a rotary evaporator and stored in the vacuum desiccator for further studies. The yield (%*w*/*w*) of the extract was calculated using the following equation [19]:

(1) $$Yield\;\left(\%\right)=\frac{Weight\;of\;extract}{Weight\;of\;dried\;powder}\times 100\%$$

### 4.2. Identification and Quantification of Bioactive Compounds in C. chinense Using High-Performance Liquid Chromatography

In the analysis of *C. chinense* stem extract, the bioactive compounds verbascoside, isoverbascoside, and hispidulin were identified and quantified. The identification and quantification were achieved through the implementation of a validated HPLC method employing an RP-C18 column (ACE 5 C18-AR, 250 × 4.6 mm, 5 μm) [42]. The mobile phase used for chromatography consisted of a mixture of acetonitrile (A) and 0.085% phosphoric acid in water (B), and a gradient elution mode was employed. Specifically, the elution conditions began with 5% eluent A and followed a linear gradient to reach 40% eluent A over the initial 20.0 min. Subsequently, another linear gradient increased the proportion of eluent A to 80% at 30.0 min and then maintained this ratio for an additional 5.0 min. The analysis was conducted at a constant flow rate of 1.0 mL/min, with an injection volume of 50 μL. The mobile phase was degassed prior to the analysis. The HPLC samples were filtered through a 0.45 μm membrane filter before analysis. Detection and quantification of the peaks were accomplished by monitoring the absorbance at 326 nm using a UV–visible detector (YL9120 UV/VIS). Identification of the peaks was achieved by comparing their retention times with those of standard verbascoside, isoverbascoside, and hispidulin. The quantification of these compounds in the extracts was achieved by constructing calibration curves and plotting the peak area against the standard concentration.

### 4.3. Formulation of C. chinense Stem Extract Nanoparticles

The preparation of *C. chinense* stem extract nanoparticles utilized the solvent displacement method. Initially, 200 mg of *C. chinense* stem extract was dissolved in 2 mL of 95% ethanol, forming the organic phase containing the extract. Afterward, the extract solution was centrifuged at 13,000 rpm for 3 min to remove any undissolved residue. Concurrently, an aqueous phase comprising 15 mL of a 0.1% poloxamer 407 solution was prepared. The organic phase was then introduced into the aqueous phase through gradual infusion at a controlled rate of 1 mL/h while maintaining continuous agitation at 700 rpm [18]. This dispersion process extended for 3 h to facilitate the evaporation of the organic solvent.

The *C. chinense* stem extract NPs were subjected to storage in different temperatures (4 °C, 30 °C, and 45 °C) and distinct time intervals (0, 0.5, 1, 2, 3, and 4 weeks). Following each incubation period, the samples were analyzed through dynamic light scattering and zeta potential measurements. The results were compared to the initial nanoparticle characterization. 

### 4.4. Determination of Total Phenolic Content (TPC) in C. chinense Stem Extract and NPs

The analysis of the total phenolic content in *C. chinense* stem extract and NPs was carried out using the Folin–Ciocalteu colorimetric method [43]. In 96-well plates, a 50 µL aliquot of either the stem extract (at concentrations of 156.25–2500 µg/mL, 100 µL per well) or the NPs (at concentrations of 104.16–1666.63 µg/mL, 100 µL per well) was combined with 100 µL of 10% *w*/*v* Folin–Ciocalteu phenol reagent. After a 4 min incubation, 50 µL of 7.5% sodium carbonate was added, and the mixture was left at room temperature for 60 min. The absorbance of the resulting solution was measured at 725 nm. A standard calibration curve using gallic acid within the concentration range of 3.9–125 μg/mL was prepared in a similar manner, and the results were expressed as milligrams of gallic acid equivalent (GAE) per gram of extract.

### 4.5. Determination of Total Flavonoid Content (TFC) in C. chinense Stem Extract and NPs

The determination of the total flavonoid contents in the extract from *C. chinense* stem extract and NPs was carried out using the aluminum chloride colorimetric method [43]. In 96-well plates, solutions of quercetin (ranging from 3.9 to 1000 µg/mL), the extract (at concentrations of 312.5–20,000 µg/mL, 100 µL per well), and NPs (at concentrations of 52.08–13,333 µg/mL, 100 µL per well) were introduced. Subsequently, 30 µL of 5% NaNO_2_ was added to each well and incubated for 5 min. This was followed by the addition of 50 µL of 2% *w*/*v* aluminum chloride and a 6 min incubation, succeeded by a 10 min incubation with 50 µL of 1 N NaOH. The absorbance was measured at 510 nm using a UV–Vis spectrophotometer (Spectramax M3, Thermo Scientific, Waltham, MA, USA). Total flavonoid contents were determined based on standard curves for quercetin, and the results were expressed as milligrams of quercetin equivalent (QE) per gram of extract.

### 4.6. Antioxidant Activities of C. chinense Stem Extract and Nanoparticles

#### 4.6.1. DPPH Free Radical Scavenging Activity Assay

The assessment of the free radical scavenging capacity of both *C. chinense* stem extract and NPs was conducted using the DPPH assay [44]. Ascorbyl glucoside served as the positive control, and it was prepared in a concentration ranging from 3.9 to 2000 μg/mL. In 96-well plates, aliquots of *C. chinense* stem extract (62.5–2000 µg/mL) and NPs (26.0–3333.33 µg/mL), each measuring 100 µL, were added. Subsequently, 100 µL of a DPPH solution at a concentration of 500 µM was introduced to the wells. The resultant mixture was incubated in darkness for a duration of 30 min, after which the absorbance was measured at 517 nm. The percentage of inhibition for both the standard and the extract was calculated at each concentration, and graphical representations were generated by plotting % inhibition against concentration [45]. The IC_50_ values were expressed as the concentration in μg/mL of the extract required to reduce the absorbance of DPPH by 50% in comparison to the negative control.

(2) $$DPPH\;Free\;radical\;scavenging\;\left(\%\right)=\frac{A-B}{A}\times 100\%$$

where *A* is the absorbance of the reaction with solvent control and *B* is the absorbance of the DPPH with the extract.

#### 4.6.2. ABTS Free Radical Scavenging Activity Assay

The ABTS radical scavenging assay was performed to confirm the free radical scavenging activity of the extract [44]. The stock solution of ABTS radical was prepared by mixing 7 mM of ABTS solution and 2.45 mM of potassium persulfate at a 1:1 ratio and allowing it to react for 12 h at room temperature in the dark. The ascorbyl glucoside (3.9–2000 µg/mL), *C. chinense* stem extract (31.25–2000 µg/mL), and NPs (26.0–6666.5 µg/mL) (20 µL) were placed in the 96-well plates, followed by adding ABTS solution (180 µL). The plates were kept in the dark for 30 min. The absorbance was read at 734 nm. The %inhibition of the standard and the extract was calculated [18]. The IC_50_ values of the standard and the sample were calculated from the graph.

(3) $$ABTS\;Free\;radical\;scavenging\;\left(\%\right)=\frac{A-B}{A}\times 100\%$$

where *A* is the absorbance of the reaction with solvent control and *B* is the absorbance of the reaction with the extract.

#### 4.6.3. Ferric-Reducing Antioxidant Power (FRAP) Assay

FRAP reagent was prepared by mixing 300 mM of acetate buffer (pH 3.6), 10 mM of 2,4,6-tripyridyl-s-triazine (TPTZ) solution in 40 mM of HCl, and 20 mM of FeCl_3_·6H_2_O solution at a 10:1:1 ratio [44]. Ascorbyl glucoside solution (3.9–2000 µg/mL) was used as the standard solution, and *C. chinense* stem extract solution (39–20,000 µg/mL) and NPs (26–13,333 µg/mL) were prepared. Samples (20 μL) were allowed to react with FRAP solution (180 μL) in 96-well plates at 37 °C for 30 min in the dark. The absorbance of ferrous tripyridyltriazine complex of the standard and the extract was read at 595 nm. The concentration of FRAP content in the extract was reported as μM Fe (II) equivalent. The FRAP content was calculated from the standard curve constructed using a ferrous sulfate solution (9.8–5000 μM).

In this study, three distinct antioxidant assays were employed, each with specific applications. The DPPH assay was utilized to evaluate the antioxidant activity of hydrophobic compounds present in both the *C. chinense* stem extract and NPs. In contrast, the ABTS assay is versatile and capable of measuring the antioxidant capacity of both hydrophilic and hydrophobic antioxidants. The FRAP assay, on the other hand, specifically measures the antioxidant activity of hydrophilic compounds in the extract and NPs. Given that the extraction solvent for the *C. chinense* stem was 95% ethanol, it facilitated the extraction of both hydrophobic and hydrophilic antioxidants.

The DPPH test is a widely employed method for assessing the antioxidant activity of plant extracts. This assay relies on the ability of antioxidants to donate electrons, thus neutralizing the DPPH radical. The ABTS test evaluates the antioxidants’ capacity to neutralize the stable radical cation ABTS^•+^. The presence of antioxidants leads to a decrease in the intensity of the blue-green chromophore. The FRAP test follows a Single Electron Transfer (SET) mechanism, measuring the reduction of ferric ions (Fe^3+^) ligand to the intensely blue ferrous complex (Fe^2+^) in acidic conditions. Antioxidants in the solution can either reduce Fe^3+^ to Fe2^+^, binding with ferricyanide to produce Prussian blue, or reduce ferricyanide to ferrocyanide, which binds free Fe^3+^ to form Prussian blue [46].

The correlations between total phenolic and total flavonoid contents and the antioxidant activities of *C. chinense* stem extract and the NPs were calculated using the Pearson correlation coefficient.

### 4.7. Cell Proliferation Assay Using a Sulforhodamine B (SRB) Assay

The anticancer activities of the *C. chinense* stem extract were tested for the first time in this study. To confirm the anticancer activity of the *C. chinense* stem extract, the cytotoxicity of the extract was tested in several cancer cell lines, including HeLa, A549, MCF-7, and SKOV-3 cells. The most sensitive cell line to this extract was further investigated for the anticancer activity mechanisms. The *C. chinense* stem extract was exposed to three distinct cancer cell lines: MCF-7, HeLa, and A549 cells. The cytotoxicity of NPs was investigated in the most sensitive cells, MCF-7 cells, and it was confirmed in A549 cells. After a 24, 48, and 72 h incubation period, the cytotoxicity of both the extract and the NPs (25, 50, 100, 250, 500, and 1000 µg/mL) was assessed via the SRB assay [19]. To fix the cells, ice-cold 10% trichloroacetic acid at 4 °C was added. Subsequently, the cells were stained with a 0.4% SRB dye for 30 min at room temperature. Removal of unbound dye was achieved through three washes with 1% acetic acid. Then, 200 µL of a 10 mm Tris base buffer was added to each well to dissolve the bound dye. The absorbance was measured using a spectrophotometer microplate reader at a wavelength of 540 nm. The percentage of cell viability was calculated by employing the following equation, with untreated cells serving as the control [19]:

(4) $$Cell\;viability\;\left(\%\right)=\frac{A540\;sample}{A540\;control}\times 100\%$$

where the A540 sample is the absorbance of treated cells and the A540 control is the absorbance of untreated cells. The IC_50_ value indicates the concentration of *C. chinense* stem extract and NPs that reduce cancer cell viability or induce cell death by 50%. The dose–response curve was created by plotting the percentage of cell viability against the log-transformed concentrations of the extract or NPs. The IC_50_ was calculated using GraphPad Prism 7.0.

*C. chinense* stem extract and NPs were treated with C2C12 normal cells, and the cell viability was measured using an SRB assay following the protocol above. The selectivity index was calculated according to the following formula: Selectivity index = (IC_50_ of a sample in SKOV-3 noncancerous cell)/(IC_50_ of a sample in cancerous cell lines).

### 4.8. Colony Formation Assay

The colony formation assay was used to assess the anchorage-dependent growth of MCF-7 cells. MCF-7 cells were seeded in 6-well plates at 2000 cells/well. Then, the cells were subjected to treatment with a concentration range of 25, 50, 100, 250, and 500 µg/mL of *C. chinense* stem extract and 50, 100, 250, 500, and 1000 µg/mL of NPs for 24 h. After a 15-day incubation period, the cells were stained with 0.5% crystal violet for 5 min at room temperature, followed by a thorough wash with deionized water and air-drying [18]. The colonies that had formed a minimum of 50 cells were observed and quantified using a light microscope at 40× magnification.

### 4.9. Annexin V/PI Apoptosis Detection Assay

Cell death mechanisms were examined using the Annexin V-FITC apoptosis detection kit (Sigma-Aldrich, St. Louis, MO, USA) by following the manufacturer’s instructions. MCF-7 cells were initially seeded in 6-well plates at a density of 2.5 × 10^5^ cells/well and left to incubate for 24 h before being subjected to treatment with *C. chinense* stem extract (100, 250, and 500 μg/mL) and NPs (250, 500, and 1000 μg/mL) for an additional 24 h. Following treatment, both the treated and untreated (negative control) cells were trypsinized and subsequently washed three times with PBS. The cells were then re-suspended in a 1× apoptosis binding buffer. To label the cells, 5 μL of Annexin V-FITC and 10 μL of PI solution were added to each suspension and allowed to incubate for 15 min at room temperature in the absence of light [18]. Cell apoptosis was assessed using a flow cytometer (BD Biosciences, San Jose, CA, USA) within 60 min, employing BD Accuri C6 Plus software version 227.4.

### 4.10. Mitochondrial Membrane Potential (MMP) Assay

The effect of *C. chinense* stem extract and NPs on mitochondrial function was determined using the JC-1 staining method. For mitochondrial function, cancer cells (2.5 × 10^5^ cells/well) were seeded into 6-well cultured plate for 24 h and treated with *C. chinense* stem extract (100, 250, and 500 µg/mL) and NPs (250, 500, and 1000 µg/mL) for 24 h. MCF-7 cells were harvested and washed, and the pellet cancer cells were collected. The cells were added to 100 µL of medium containing 5 µL of JC-1 assay reagents for 30 min at 37 °C in the dark; finally, 400 µL of DMEM medium was added. The mitochondrial membrane potential was determined through flow cytometric analysis (BD Biosciences, CA, USA) using BD Accuri C6 Plus software.

### 4.11. Cell Cycle Arrest Assay

Flow cytometry was used to investigate how *C. chinense* stem extract and NPs affect the cell cycle distribution of MCF-7 cells [18]. The cells, initially seeded at a density of 2.5 × 10^5^ cells/mL, were cultured in 6-well plates for 24 h before exposure to *C. chinense* stem extract (100, 250, and 500 µg/mL) and NPs (250, 500, and 1000 µg/mL) for an additional 24 h incubation. Following trypsinization, the cells were washed with PBS buffer and then fixed with ice-cold 70% ethanol at −20 °C. Subsequently, the cells were treated with PI solution (BD Biosciences, CA, USA) for 30 min at 4 °C. Flow cytometry, with the aid of BD Accuri C6 Plus software, was employed to analyze the cell cycle phases, and the resulting fluorescent signals were presented as histograms. Gated cells were manually classified into their respective cell cycle stages.

### 4.12. Statistical Analysis

The data were expressed as mean ± S.D. Statistical analysis was conducted using one-way analysis of variance (ANOVA) and Tukey’s post hoc test. Additionally, a *t*-test was employed to assess the significance of the difference between the means of the two groups. A *p*-value < 0.05 was deemed statistically significant.

## 5. Conclusions

This study explored the cytotoxic impact of *C. chinense* stem extract on four human cancer cell lines, including MCF-7, A549, HeLa, and SKOV-3 cells. The MCF-7 cell line was mostly sensitive to the extract with the highest selectivity index. While nanoparticles derived from the extract exhibited reduced cytotoxicity against MCF-7 cells, they did not disrupt the extract’s anticancer mechanism. Investigations into the underlying mechanisms revealed that both the *C. chinense* stem extract and its nanoparticles significantly impeded the growth of MCF-7 cells by inducing apoptosis, inhibiting colony formation, reducing mitochondrial membrane potential, and causing cell cycle arrest through the regulation of the G0/G1 transition.

## Figures and Tables

**Table 1:** Pearson correlation coefficients of total phenolic and flavonoid contents and antioxidant activities of *C. chinense* stem ethanolic extract and NPs measured using DPPH, ABTS, and FRAP assays.

Assay	Total Phenolic Content (Gallic Acid Equivalent)		Total Flavonoid Content (Quercetin Equivalent)	
	*C. chinense* Extract	*C. chinense* Extract NPs	*C. chinense* Extract	*C. chinense* Extract NPs
DPPH assay	0.9515 *	0.9996 ****	0.8734	0.9982 ****
ABTS assay	0.9740 **	0.9824 ****	0.9997 ****	0.8877 *
FRAP assay	0.9789 **	0.9992 ****	0.9998 ****	0.9983 ****

* indicates *p* < 0.05, ** indicates *p* < 0.01, and **** indicates *p* < 0.0001.

**Table 2:** IC_50_ values and selectivity index of *C. chinense* stem extract and NPs against cancerous and non-cancerous cells.

	IC_50_ Value			Selectivity Index		
	24 h	48 h	72 h	24 h	48 h	72 h
*C. chinense* stem extract						
MCF-7	430.4	210.3	109.2	1.3	1.7	3.0
HeLa	499.4	213.5	155.6	1.1	1.7	2.1
A549	733.4	320.5	206.9	0.8	1.1	1.6
SKOV-3	1501	486.4	423	0.4	0.7	0.8
C2CL12	558.7	355.3	329.2	-	-	-
*C. chinense* stem extract NPs						
MCF-7	2329	2150	1664	4.1	1.3	1.1
A549	4960	2673	1363	1.9	1.0	1.4
SKOV-3	10,201	3079	2044	0.9	0.9	0.9
C2CL12	9528	2799	1905	-	-	-

**Table 3:** Analysis of cell apoptosis induced by *C. chinense* stem extract and NPs.

Groups of Treatment	Viable Cells (%)	Late Apoptosis (%)	Necrosis (%)
Control	93.30 ± 0.26	3.27 ± 0.17	2.17 ± 0.03
Extract 100 µg/mL	52.43 ± 0.07 *	14.07 ± 0.57 *	31.30 ± 0.66 *
Extract 250 µg/mL	56.97 ± 0.58 *	14.77 ± 0.44 *	27.17 ± 0.80 *
Extract 500 µg/mL	16.13 ± 0.50 *	12.13 ± 0.80 *	71.53 ± 1.27 *
NPs 250 µg/mL	39.57 ± 0.87 *	26.73 ± 3.89 *	32.23 ± 4.73 *
NPs 500 µg/mL	14.27 ± 0.26 *	25.57 ± 0.57 *	59.30 ± 0.81 *
NPs 1000 µg/mL	6.10 ± 0.12 *	40.13 ± 1.00 *	52.07 ± 0.83 *

The data are expressed in terms of mean ± S.D. from three independent experiments. * indicates *p* < 0.05. Tukey’s multiple comparisons test for apoptosis statistical analysis are shown in Supplementary Table S5.

**Table 4:** Percentage of JC-1 aggregates, JC-1 monomers, and ratio of monomers to aggregate MCF-7 cells treated with *C. chinense* stem extract and NPs.

Groups of Treatment	JC-1 Aggregates	JC-1 Monomers	Ratio of Monomers to Aggregates
Control	91.07 ± 0.19	6.07 ± 0.22	0.067
Extract 100 µg/mL	92.47 ± 0.35	5.83 ± 0.34	0.063
Extract 250 µg/mL	87.27 ± 3.03 *	10.67 ± 2.75 *	0.130
Extract 500 µg/mL	79.87 ± 4.82 *	15.97 ± 4.09 *	0.200
NPs 250 µg/mL	82.27 ± 3.82 *	15.23 ± 3.82 *	0.185
NPs 500 µg/mL	77.97 ± 0.61 *	18.17 ± 0.38 *	0.233
NPs 1000 µg/mL	38.37 ± 1.53 *	52.90 ± 1.10 *	1.379

The data are expressed in terms of mean ± S.D. from three independent experiments. * indicates *p* value < 0.05. Tukey’s multiple comparisons test for mitochondrial membrane potential statistical analysis are shown in Supplementary Table S6.

**Table 5:** The percentage of MCF-7 cells treated with *C. chinense* stem extract and NPs in each phase of the cell cycles.

Groups of Treatment	G0/G1 Phase	S Phase	G2/M Phase
Control	64.63 ± 0.44	15.57 ± 0.24	17.53 ± 0.55
Extract 100 µg/mL	66.53 ± 0.23 *	14.97 ± 0.03	15.97 ± 0.19 *
Extract 250 µg/mL	66.57 ± 0.24 *	19.50 ± 0.15 *	10.87 ± 0.09 *
Extract 500 µg/mL	71.40 ± 0.56 *	15.97 ± 0.34	8.90 ± 0.26 *
NPs 250 µg/mL	66.57 ± 0.38 *	13.87 ± 0.13 *	15.47 ± 0.30 *
NPs 500 µg/mL	74.63 ± 0.28 *	11.77 ± 0.52 *	9.63 ± 0.09 *
NPs 1000 µg/mL	67.87 ± 0.03 *	13.50 ± 0.15 *	11.77 ± 0.29 *

The data are shown as means ± S.D. for three independent experiments. * indicates *p* < 0.05. Tukey’s multiple comparisons test for cell cycle arrest statistical analysis are shown in Supplementary Table S7.

## Data Availability

Data is contained within the article and Supplementary Material.

## Supplementary Materials

The following supporting information can be downloaded at: https://www.mdpi.com/article/10.3390/ijms25020978/s1.

## Abbreviations

A549	Human lung carcinoma
ABTS	2,2′-Azino-bis(3-ethylbenzothiazoline-6-sulfonic acid)
ANOVA	Analysis of variance
DPPH	2,2-Diphenyl-1-picrylhydrazyl
EGCG	Epigallocatechin gallate
FRAP	Ferric-Reducing Antioxidant Power
GAE	Gallic acid equivalent
HeLa	Human epithelial cervical cancer
HPLC	High-performance liquid chromatography
JC-1	5,5,6,6-Tetrachloro-1,1,3,3-tetraethylbenzimidazolylcarbocyanine iodide
MCF-7	Human breast adenocarcinoma
MMP	Mitochondrial membrane potential
NAPIs	Natural active pharmaceutical ingredients
NPs	Nanoparticles
PDI	Polydispersity index
QE	Quercetin equivalent
RP	Reversed Phase
SRB	Sulforhodamine B
TEM	Transmission electron microscopy
TFC	Total flavonoid content
TPC	Total phenolic content
ZTP	Ziyang green tea
