# Supplementary material for: Clerodendrum chinense Stem Extract and Nanoparticles: Effects on Proliferation, Colony Formation, Apoptosis Induction, Cell Cycle Arrest, and Mitochondrial Membrane Potential in Human Breast Adenocarcinoma Breast Cancer Cells
Source: Int J Mol Sci. 2024 Jan 12;25(2):978. doi: 10.3390/ijms25020978 (PMC10815711; doi:10.3390/ijms25020978)
Supplement: Supplementary file 1 [file ijms-25-00978-s001.zip › ijms-2765607-supplementary.pdf]

Supplementary data of a manuscript entitled *Clerodendrum chinense* Stem Extract and Nanoparticles: Effects on Proliferation, Colony Formation, Apoptosis Induction, Cell Cycle Arrest, and MMP in MCF-7 Breast Cancer Cells

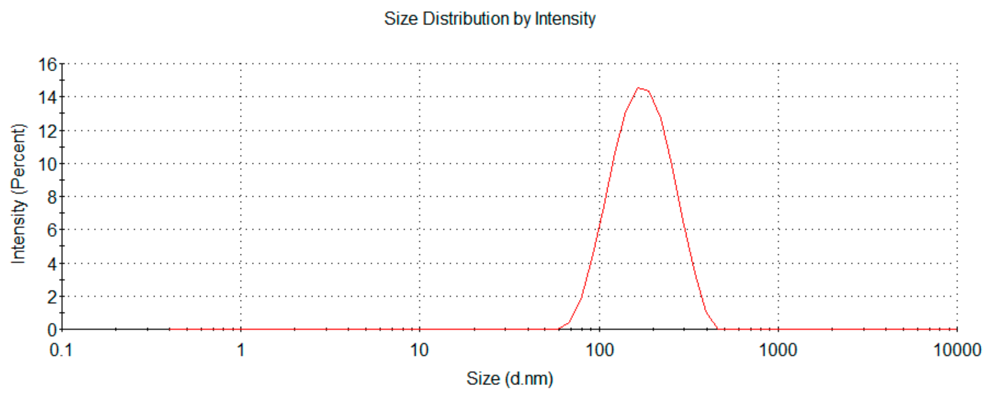

Supplementary Figure S1 Particle size distribution analysis of *C. chinense* stem extract

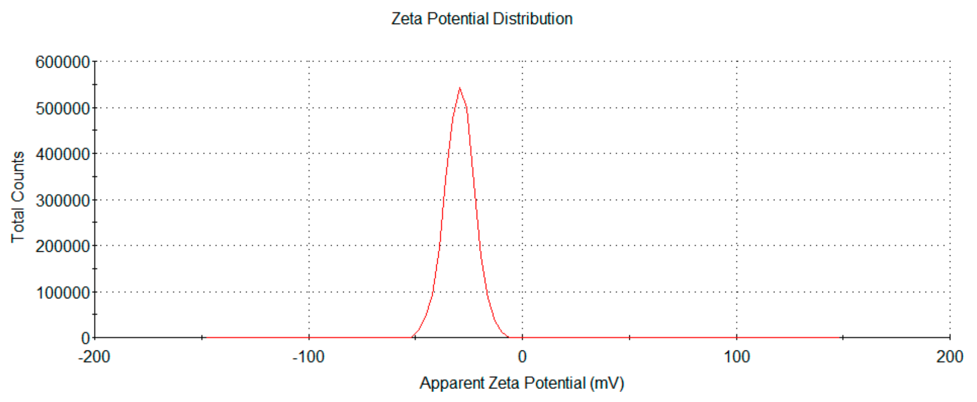

Supplementary Figure S2 Zeta potential analysis of *C. chinense* stem extract

A

			Size (d.nm):	% Intensity:	St Dev (d.nm):
Z-Average (d.nm):	190.3	Peak 1:	223.4	100.0	82.33
PdI:	0.136	Peak 2:	0.000	0.0	0.000
Intercept:	0.918	Peak 3:	0.000	0.0	0.000

Result quality : Good

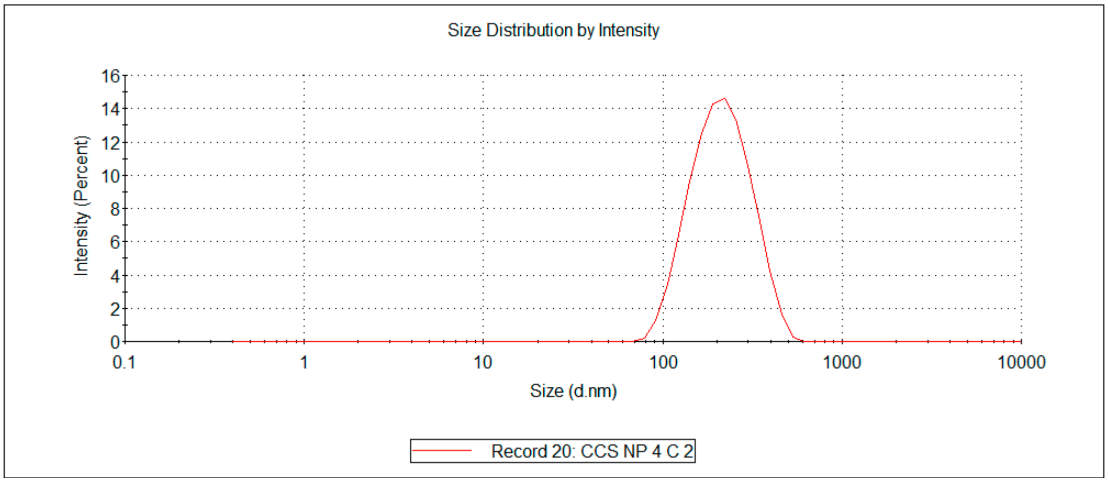

B

			Mean (mV)	Area (%)	St Dev (mV)
Zeta Potential (mV):	-24.9	Peak 1:	-24.9	100.0	3.23
Zeta Deviation (mV):	3.23	Peak 2:	0.00	0.0	0.00
Conductivity (mS/cm):	0.0514	Peak 3:	0.00	0.0	0.00

Result quality : Good

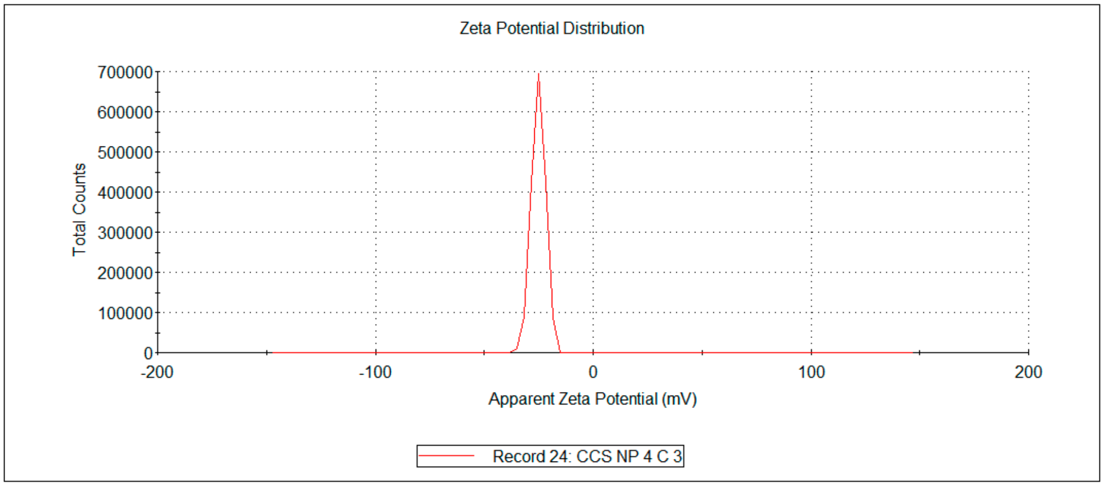

C

			Size (d.nm):	% Intensity:	St Dev (d.nm):
Z-Average (d.nm):	170.5	Peak 1:	203.7	100.0	92.46
PdI:	0.152	Peak 2:	0.000	0.0	0.000
Intercept:	0.910	Peak 3:	0.000	0.0	0.000

Result quality : Good

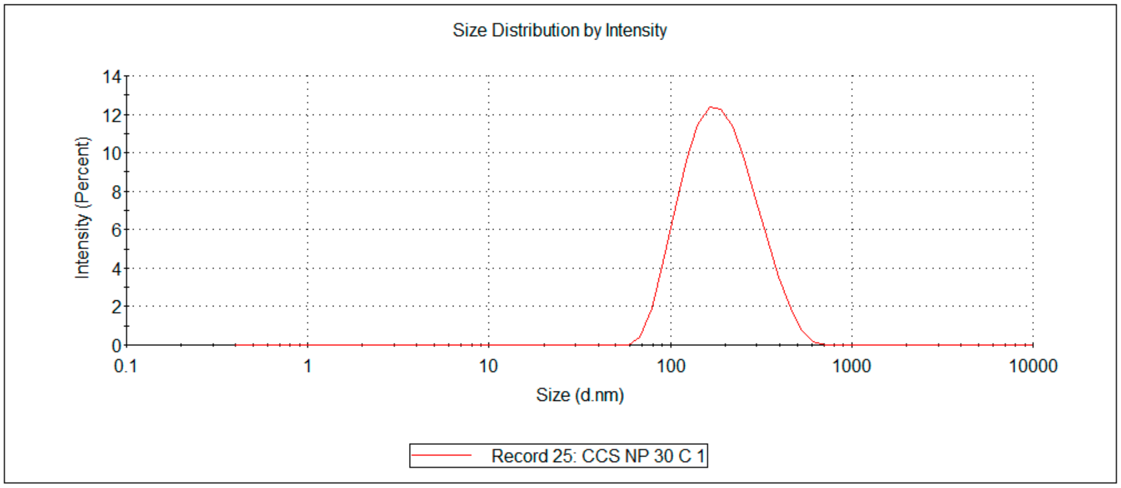

D

			Mean (mV)	Area (%)	St Dev (mV)
Zeta Potential (mV):	-31.6	Peak 1:	-31.6	100.0	7.07
Zeta Deviation (mV):	7.07	Peak 2:	0.00	0.0	0.00
Conductivity (mS/cm):	0.0476	Peak 3:	0.00	0.0	0.00

Result quality : Good

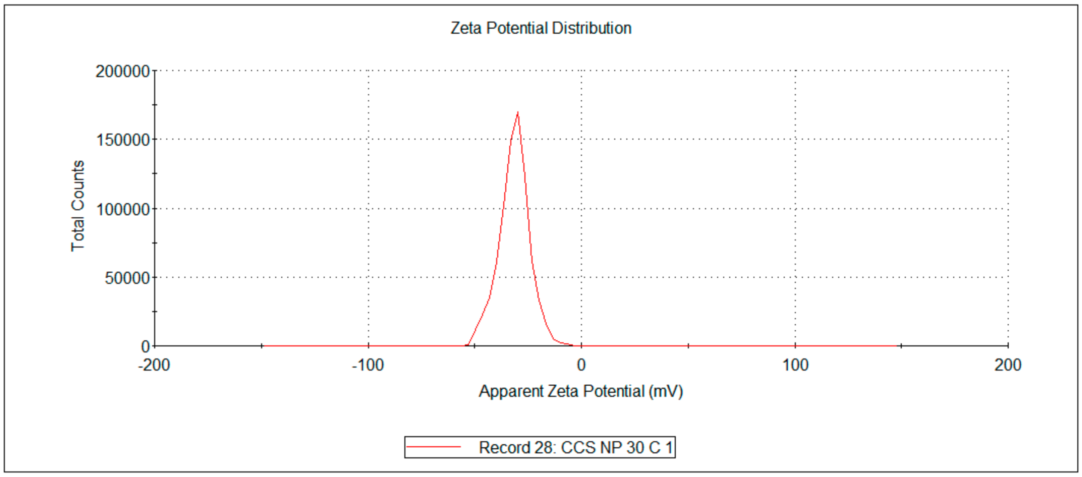

E

			Size (d.nm):	% Intensity:	St Dev (d.nm):
Z-Average (d.nm):	151.7	Peak 1:	164.7	100.0	48.63
PdI:	0.071	Peak 2:	0.000	0.0	0.000
Intercept:	0.930	Peak 3:	0.000	0.0	0.000
Result quality :	Good				

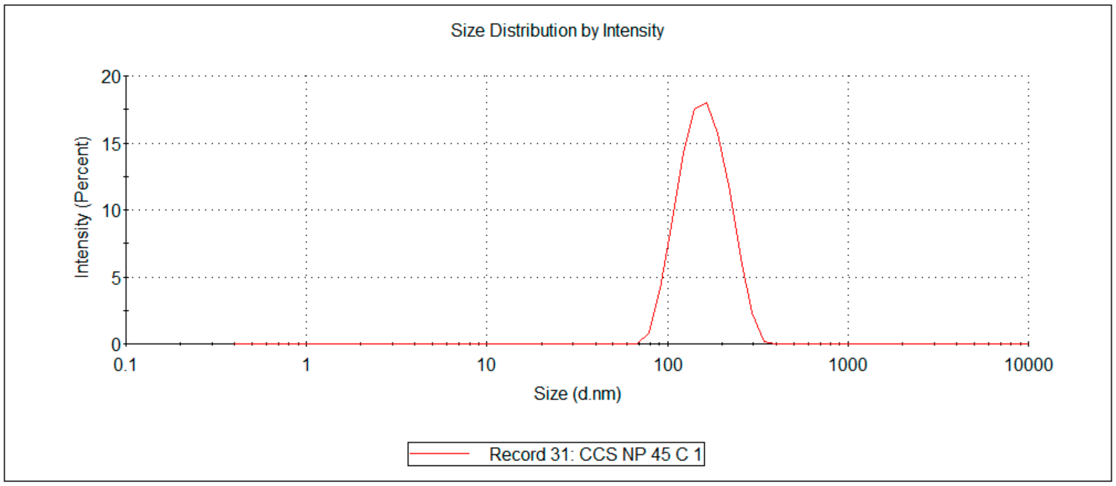

F

			Mean (mV)	Area (%)	St Dev (mV)
Zeta Potential (mV):	-27.6	Peak 1:	-27.6	100.0	7.68
Zeta Deviation (mV):	7.68	Peak 2:	0.00	0.0	0.00
Conductivity (mS/cm):	0.0655	Peak 3:	0.00	0.0	0.00
Result quality :	Good				

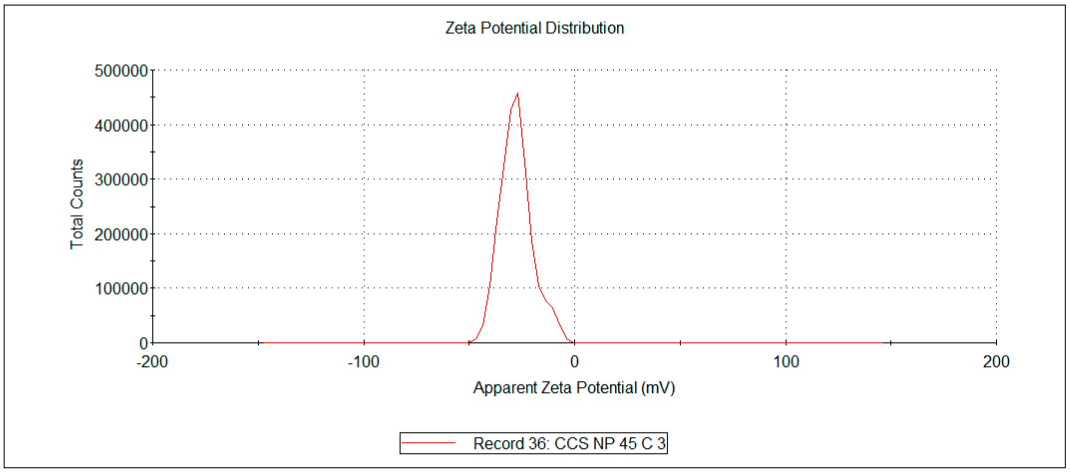

Supplementary Figure S3 Size of *C. chinense* stem extract NPs stored at (A) 4, (B) 30, and (C) 45 °C for 4 months and zeta potential values of *C. chinense* stem extract NPs stored at (D) 4, (E) 30, and (F) 45 °C for 4 months.

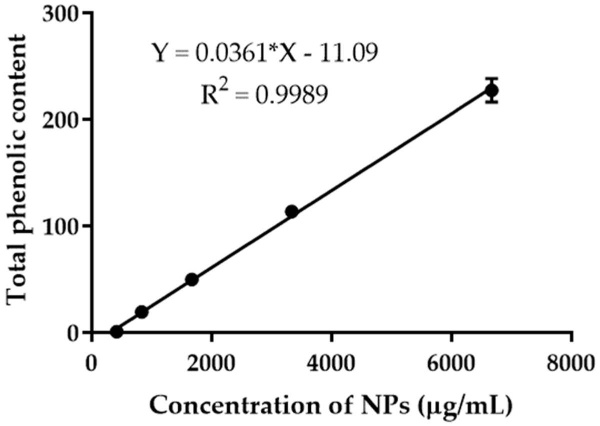

Supplementary Figure S4 Plots of total phenolic contents and *C. chinense* stem extract concentrations

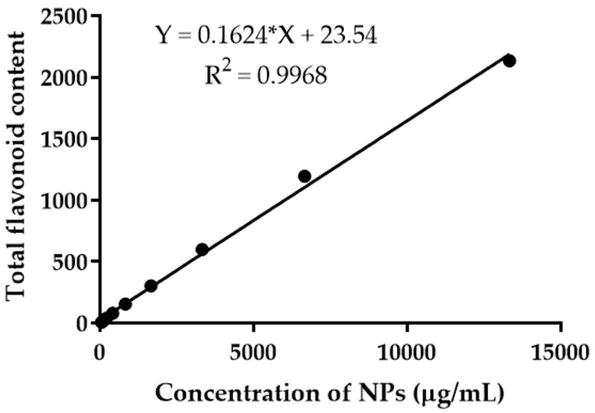

Supplementary Figure S5 Plots of total flavonoid contents and *C. chinense* stem extract concentrations

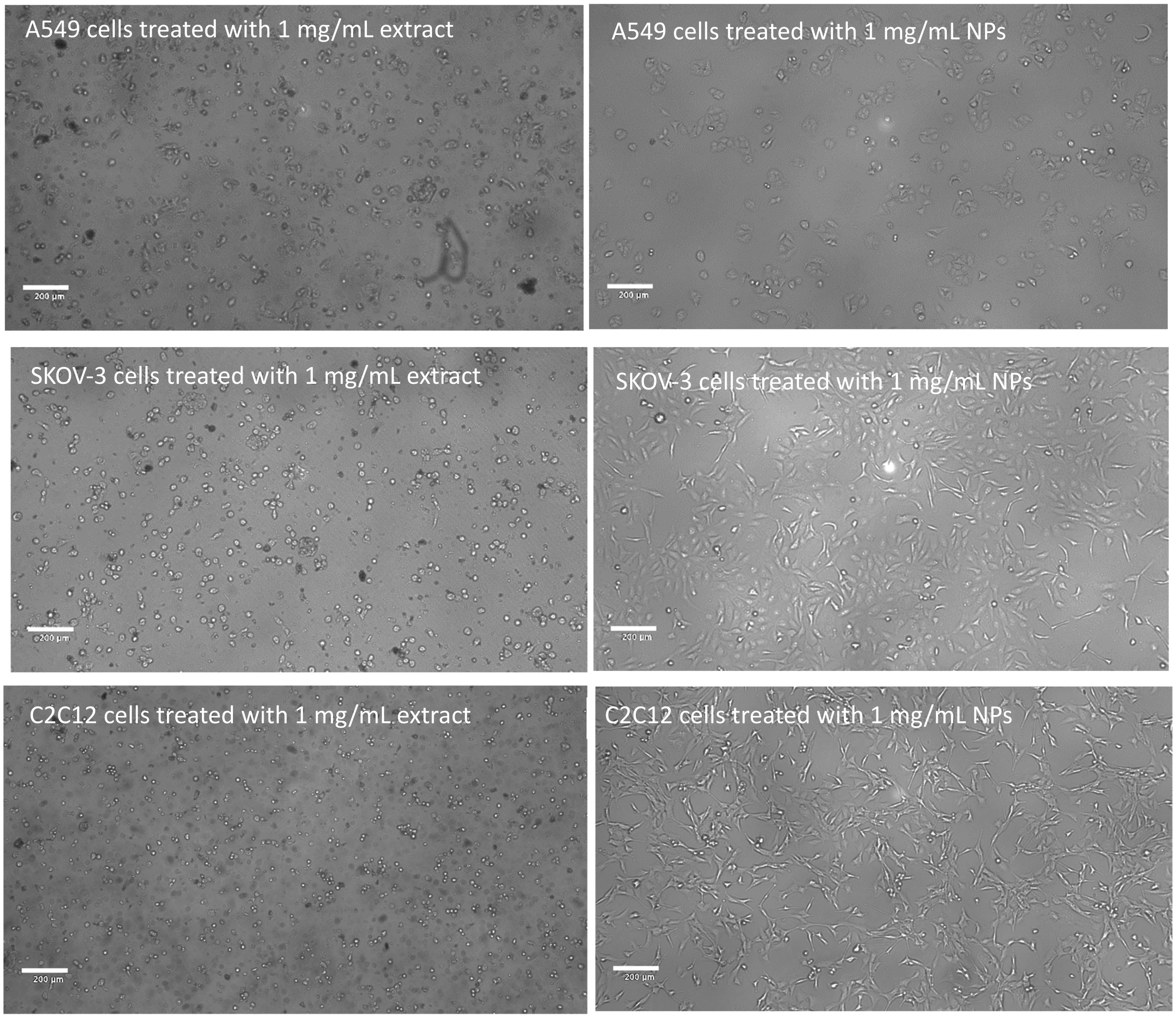

Supplementary Figure S6 Micrographs of cells treated with *C. chinense* stem extract or NPs at 1 mg/mL for 24 h.

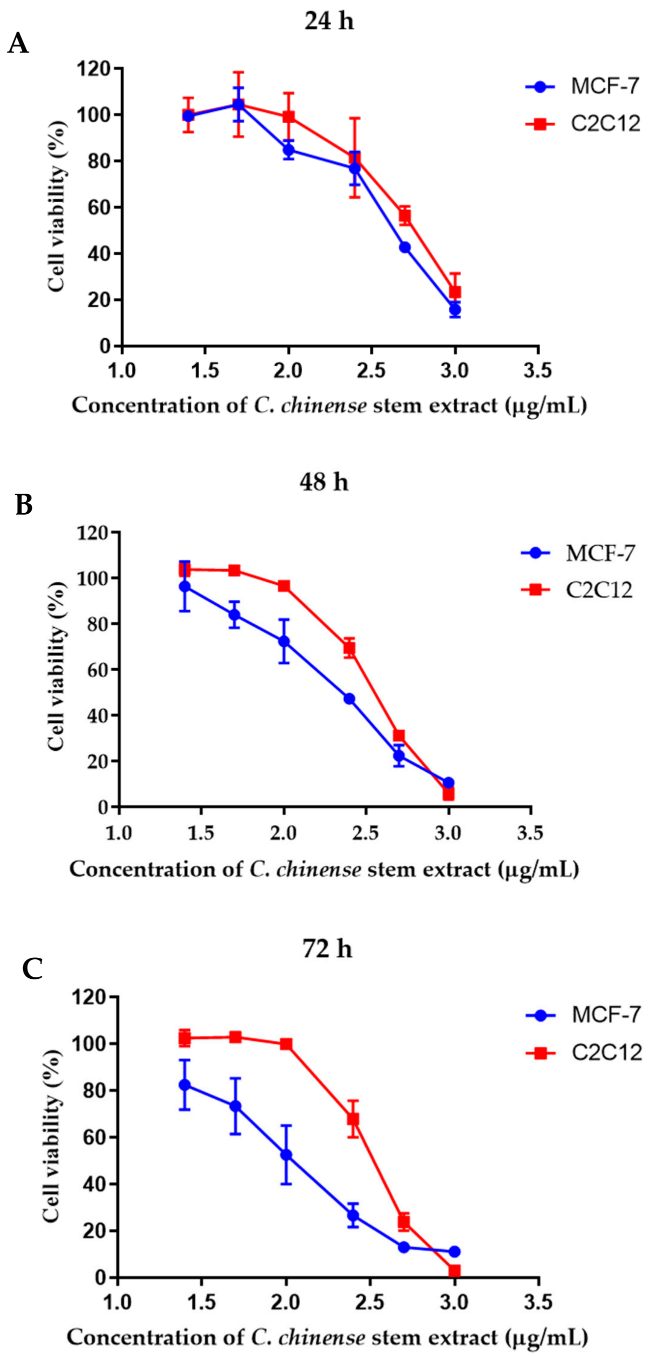
A
24 h
MCF-7
C2C12
Cell viability (%)
0
20
40
60
80
100
120
1.0
1.5
2.0
2.5
3.0
3.5
Concentration of *C. chinense* stem extract (µg/mL)
B
48 h
MCF-7
C2C12
Cell viability (%)
0
20
40
60
80
100
120
1.0
1.5
2.0
2.5
3.0
3.5
Concentration of *C. chinense* stem extract (µg/mL)
C
72 h
MCF-7
C2C12
Cell viability (%)
0
20
40
60
80
100
120
1.0
1.5
2.0
2.5
3.0
3.5
Concentration of *C. chinense* stem extract (µg/mL)

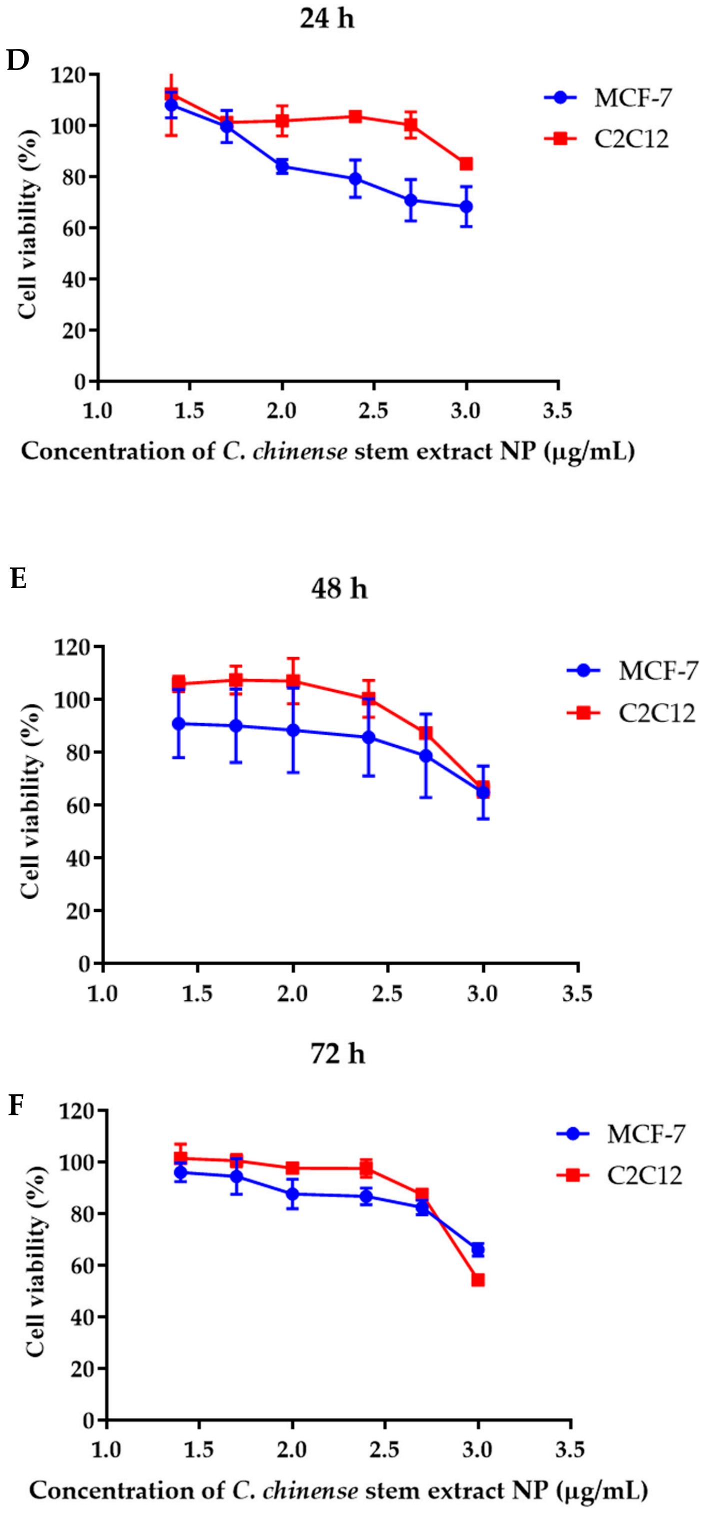

Supplementary Figure S7 Cell viability and growth of MCF-7 breast cancer cells and C2C12 myoblast cells after treatment with *C. chinense* stem extract for (A) 24 h, (B) 48 h, (C) 72 h and the NPs for (D) 24 h, (E) 48 h, and (F) 72 h. Data were presented as the mean ± SD of the three independent experiments.

Supplementary Table S1 Tukey's multiple comparisons test for particle size statistical analysis

4 °C	Mean Diff.	95.00% CI of diff.	Significant?	Summary	Adjusted P Value
0 vs. 0.5	-38.73	-46.14 to -31.32	Yes	****	<0.0001
0 vs. 1	-27.43	-34.84 to -20.02	Yes	****	<0.0001
0 vs. 2	-18.17	-25.58 to -10.76	Yes	****	<0.0001
0 vs. 3	-19.73	-27.14 to -12.32	Yes	****	<0.0001
0 vs. 4	-30.27	-37.68 to -22.86	Yes	****	<0.0001

30 °C	Mean Diff.	95.00% CI of diff.	Significant?	Summary	Adjusted P Value
0 vs. 0.5	-6.067	-13.48 to 1.342	No	ns	0.1622
0 vs. 1	-8.567	-15.98 to -1.158	Yes	*	0.0156
0 vs. 2	-2.233	-9.642 to 5.175	No	ns	0.9422
0 vs. 3	-3.067	-10.48 to 4.342	No	ns	0.8116
0 vs. 4	-7.467	-14.88 to -0.05813	Yes	*	0.0473

45 °C	Mean Diff.	95.00% CI of diff.	Significant?	Summary	Adjusted P Value
0 vs. 0.5	-33.07	-40.48 to -25.66	Yes	****	<0.0001
0 vs. 1	-16	-23.41 to -8.591	Yes	****	<0.0001
0 vs. 2	2.933	-4.475 to 10.34	No	ns	0.8381
0 vs. 3	-3.833	-11.24 to 3.575	No	ns	0.6311
0 vs. 4	11.17	3.758 to 18.58	Yes	***	0.0008

Supplementary Table S2 Tukey's multiple comparisons test for polydispersity index statistical analysis

4 °C	Mean Diff.	95.00% CI of diff.	Significant?	Summary	Adjusted P Value
0 vs. 0.5	-0.1527	-0.2121 to -0.09325	Yes	****	<0.0001
0 vs. 1	-0.1117	-0.1711 to -0.05225	Yes	****	<0.0001
0 vs. 2	-0.064	-0.1234 to -0.004584	Yes	*	0.0285
0 vs. 3	-0.066	-0.1254 to -0.006584	Yes	*	0.0221
0 vs. 4	-0.098	-0.1574 to -0.03858	Yes	***	0.0002

30 °C	Mean Diff.	95.00% CI of diff.	Significant?	Summary	Adjusted P Value
0 vs. 0.5	-0.053	-0.1124 to 0.006416	No	ns	0.1036
0 vs. 1	-0.048	-0.1074 to 0.01142	No	ns	0.1729
0 vs. 2	-0.026	-0.08542 to 0.03342	No	ns	0.7741
0 vs. 3	-0.06467	-0.1241 to -0.00525	Yes	*	0.0262
0 vs. 4	-0.06533	-0.1247 to -0.005917	Yes	*	0.0241

45 °C	Mean Diff.	95.00% CI of diff.	Significant?	Summary	Adjusted P Value
0 vs. 0.5	-0.102	-0.1614 to -0.04258	Yes	***	0.0001
0 vs. 1	-0.08767	-0.1471 to -0.02825	Yes	**	0.0011
0 vs. 2	-0.01833	-0.07775 to 0.04108	No	ns	0.9365
0 vs. 3	-0.014	-0.07342 to 0.04542	No	ns	0.9797
0 vs. 4	0.003334	-0.05608 to 0.06275	No	ns	>0.9999

Supplementary Table S3 Tukey's multiple comparisons test for zeta potential statistical analysis

4 °C	Mean Diff.	95.00% CI of diff.	Significant?	Summary	Adjusted P Value
0 vs. 0.5	1.2	-2.18 to 4.58	No	ns	0.8907
0 vs. 1	-3.167	-6.547 to 0.2132	No	ns	0.0772
0 vs. 2	-3.367	-6.747 to 0.01323	No	ns	0.0514
0 vs. 3	-3.333	-6.713 to 0.04653	No	ns	0.0551
0 vs. 4	-3.167	-6.547 to 0.2132	No	ns	0.0772

30 °C	Mean Diff.	95.00% CI of diff.	Significant?	Summary	Adjusted P Value
0 vs. 0.5	1.267	-2.113 to 4.647	No	ns	0.8667
0 vs. 1	0.3666	-3.013 to 3.747	No	ns	0.9995
0 vs. 2	-0.7	-4.08 to 2.68	No	ns	0.9886
0 vs. 3	1.367	-2.013 to 4.747	No	ns	0.826
0 vs. 4	1.867	-1.513 to 5.247	No	ns	0.5649

45 °C	Mean Diff.	95.00% CI of diff.	Significant?	Summary	Adjusted P Value
0 vs. 0.5	4.167	0.7867 to 7.547	Yes	**	0.0085
0 vs. 1	0.9666	-2.413 to 4.347	No	ns	0.9534
0 vs. 2	-2.567	-5.947 to 0.8132	No	ns	0.2266
0 vs. 3	-1.233	-4.613 to 2.147	No	ns	0.879
0 vs. 4	-0.7334	-4.113 to 2.647	No	ns	0.9859

Supplementary Table S4 Tukey's multiple comparisons test for colony formation percentage statistical analysis

CCS	Mean Diff.	95.00% CI of diff.	Significant?	Summary	Adjusted P Value
0 vs. 25	5.95	-6.945 to 18.84	No	ns	0.7111
0 vs. 50	9.09	-3.805 to 21.98	No	ns	0.2832
0 vs. 100	45.98	33.09 to 58.87	Yes	****	<0.0001
0 vs. 250	78.67	65.78 to 91.56	Yes	****	<0.0001
0 vs. 500	98.6	85.71 to 111.5	Yes	****	<0.0001

CCS NP	Mean Diff.	95.00% CI of diff.	Significant?	Summary	Adjusted P Value
0 vs. 25	19.19	6.295 to 32.08	Yes	**	0.0014
0 vs. 50	25.19	12.3 to 38.08	Yes	****	<0.0001
0 vs. 100	86.24	73.35 to 99.13	Yes	****	<0.0001
0 vs. 250	100	87.11 to 112.9	Yes	****	<0.0001
0 vs. 500	100	87.11 to 112.9	Yes	****	<0.0001

Supplementary Table 5 Tukey's multiple comparisons test for apoptosis statistical analysis

C. chinense leaf extract

Live	Mean Diff.	95.00% CI of diff.	Significant?	Summary	Adjusted P Value
0 vs. 100	40.87	34.43 to 47.31	Yes	****	<0.0001
0 vs. 250	36.33	29.89 to 42.77	Yes	****	<0.0001
0 vs. 500	77.17	70.73 to 83.61	Yes	****	<0.0001

late apoptosis	Mean Diff.	95.00% CI of diff.	Significant?	Summary	Adjusted P Value
0 vs. 100	-10.8	-17.24 to -4.357	Yes	***	0.0001
0 vs. 250	-11.5	-17.94 to -5.057	Yes	****	<0.0001
0 vs. 500	-8.86	-15.3 to -2.417	Yes	**	0.002

Necrosis	Mean Diff.	95.00% CI of diff.	Significant?	Summary	Adjusted P Value
0 vs. 100	-29.13	-35.57 to -22.69	Yes	****	<0.0001
0 vs. 250	-25	-31.44 to -18.56	Yes	****	<0.0001
0 vs. 500	-69.36	-75.8 to -62.92	Yes	****	<0.0001

C. chinense leaf extract NPs

Live	Mean Diff.	95.00% CI of diff.	Significant?	Summary	Adjusted P Value
0 vs. 250	53.73	47.29 to 60.17	Yes	****	<0.0001
0 vs. 500	79.03	72.59 to 85.47	Yes	****	<0.0001
0 vs. 1000	87.2	80.76 to 93.64	Yes	****	<0.0001

late apoptosis	Mean Diff.	95.00% CI of diff.	Significant?	Summary	Adjusted P Value
0 vs. 250	-23.46	-29.9 to -17.02	Yes	****	<0.0001
0 vs. 500	-22.3	-28.74 to -15.86	Yes	****	<0.0001
0 vs. 1000	-36.86	-43.3 to -30.42	Yes	****	<0.0001

Necrosis	Mean Diff.	95.00% CI of diff.	Significant?	Summary	Adjusted P Value

0 vs. 250	-30.06	-36.5 to -23.62	Yes	****	<0.0001
0 vs. 500	-57.13	-63.57 to -50.69	Yes	****	<0.0001
0 vs. 1000	-49.9	-56.34 to -43.46	Yes	****	<0.0001

Supplementary Table S6 Sidak's multiple comparisons test for Mitochondrial Membrane Potential (MMP) statistical analysis

JC-1 aggregates

CCS	Mean Diff.	95.00% CI of diff.	Significant?	Summary	Adjusted P Value
control agg vs. 100	-1.36	-11.96 to 9.241	No	ns	0.9993
control agg vs. 250	3.8	-6.801 to 14.4	No	ns	0.8808
control agg vs. 500	11.2	0.5985 to 21.8	Yes	*	0.0353

CCS NP	Mean Diff.	95.00% CI of diff.	Significant?	Summary	Adjusted P Value
control agg vs. 100	9.23	-1.371 to 19.83	No	ns	0.1084
control agg vs. 250	13.53	2.929 to 24.13	Yes	**	0.0089
control agg vs. 500	53.13	42.53 to 63.73	Yes	****	<0.0001

JC-1 monomers

CCS	Mean Diff.	95.00% CI of diff.	Significant?	Summary	Adjusted P Value
control mono vs. 100	0.24	-9.295 to 9.775	No	ns	>0.9999
control mono vs. 250	-4.6	-14.14 to 4.935	No	ns	0.6668
control mono vs. 500	-9.9	-19.44 to -0.3649	Yes	*	0.0395

CCS NP	Mean Diff.	95.00% CI of diff.	Significant?	Summary	Adjusted P Value
control mono vs. 100	-9.2	-18.74 to 0.3351	No	ns	0.0619
control mono vs. 250	-12.14	-21.68 to -2.605	Yes	**	0.0091
control mono vs. 500	-46.87	-56.41 to -37.33	Yes	****	<0.0001

Supplementary Table S7 Tukey's multiple comparisons test for cell cycle arrest statistical analysis

C. chinense stem extract

G0/G1	Mean Diff.	95.00% CI of diff.	Significant?	Summary	Adjusted P Value
0 vs. 100	-1.9	-3.149 to -0.6511	Yes	**	0.0017
0 vs. 250	-1.94	-3.189 to -0.6911	Yes	**	0.0014
0 vs. 500	-6.77	-8.019 to -5.521	Yes	****	<0.0001

S	Mean Diff.	95.00% CI of diff.	Significant?	Summary	Adjusted P Value
0 vs. 100	0.6	-0.6489 to 1.849	No	ns	0.5564
0 vs. 250	-3.93	-5.179 to -2.681	Yes	****	<0.0001
0 vs. 500	-0.4	-1.649 to 0.8489	No	ns	0.8133

G2/M	Mean Diff.	95.00% CI of diff.	Significant?	Summary	Adjusted P Value
0 vs. 100	1.56	0.3111 to 2.809	Yes	*	0.0106
0 vs. 250	6.66	5.411 to 7.909	Yes	****	<0.0001
0 vs. 500	8.63	7.381 to 9.879	Yes	****	<0.0001

C. chinense stem extract NPs

G0/G1	Mean Diff.	95.00% CI of diff.	Significant?	Summary	Adjusted P Value
0 vs. 100	-3.54	-4.99 to -2.09	Yes	****	<0.0001
0 vs. 250	-11.6	-13.05 to -10.15	Yes	****	<0.0001
0 vs. 500	-4.84	-6.29 to -3.39	Yes	****	<0.0001

S	Mean Diff.	95.00% CI of diff.	Significant?	Summary	Adjusted P Value
0 vs. 100	3.33	1.88 to 4.78	Yes	****	<0.0001
0 vs. 250	5.43	3.98 to 6.88	Yes	****	<0.0001
0 vs. 500	3.7	2.25 to 5.15	Yes	****	<0.0001

G2/M	Mean Diff.	95.00% CI of diff.	Significant?	Summary	Adjusted P Value
0 vs. 100	2.6	1.15 to 4.05	Yes	***	0.0003
0 vs. 250	8.44	6.99 to 9.89	Yes	****	<0.0001
0 vs. 500	6.3	4.85 to 7.75	Yes	****	<0.0001
